# Understanding the opioid syndemic in North Carolina: A novel approach to modeling and identifying factors

**DOI:** 10.1093/biostatistics/kxae052

**Published:** 2025-01-27

**Authors:** Eva Murphy, David Kline, Kathleen L Egan, Kathryn E Lancaster, William C Miller, Lance A Waller, Staci A Hepler

**Affiliations:** Department of Statistical Sciences, College of Arts and Sciences, Wake Forest University, 127 Manchester Hall, Winston-Salem, NC, 27109, United States; Division of Public Health Sciences, Department of Biostatistics and Data Science, Wake Forest University School of Medicine, 475 Vine Street, Winston-Salem, NC, 27101, United States; Division of Public Health Sciences, Department of Implementation Science, Wake Forest University School of Medicine, 475 Vine Street, Winston-Salem, NC, 27101, United States; Division of Public Health Sciences, Department of Implementation Science, Wake Forest University School of Medicine, 475 Vine Street, Winston-Salem, NC, 27101, United States; Gillings School of Global Public Health, University of North Carolina Chapel Hill, 135 Dauer Drive, Chapel Hill, NC, 27599, United States; Department of Biostatistics and Bioinformatics, Rollins School of Public Health, Emory University, 1518 Clifton Rd. NE, Atlanta, GA, 30322, United States; Department of Statistical Sciences, College of Arts and Sciences, Wake Forest University, 127 Manchester Hall, Winston-Salem, NC, 27109, United States

**Keywords:** Bayesian, factor analysis, identification, LQ decomposition, opioid misuse, syndemic

## Abstract

The opioid epidemic is a significant public health challenge in North Carolina, but limited data restrict our understanding of its complexity. Examining trends and relationships among different outcomes believed to reflect opioid misuse provides an alternative perspective to understand the opioid epidemic. We use a Bayesian dynamic spatial factor model to capture the interrelated dynamics within six different county-level outcomes, such as illicit opioid overdose deaths, emergency department visits related to drug overdose, treatment counts for opioid use disorder, patients receiving prescriptions for buprenorphine, and newly diagnosed cases of acute and chronic hepatitis C virus and human immunodeficiency virus. We design the factor model to yield meaningful interactions among predefined subsets of these outcomes, causing a departure from the conventional lower triangular structure in the loadings matrix and leading to familiar identifiability issues. To address this challenge, we propose a novel approach that involves decomposing the loadings matrix within a Markov chain Monte Carlo algorithm, allowing us to estimate the loadings and factors uniquely. As a result, we gain a better understanding of the spatio-temporal dynamics of the opioid epidemic in North Carolina.

## 1. INTRODUCTION

Illicit drug use in the United States has evolved over the past two decades, transitioning from predominantly misuse of prescription drugs to heroin with the recent addition of fentanyl. This transition has led to epidemic levels of drug use in many parts of the country (United States Drug Enforcement Administration 2020). The opioid epidemic is considered a syndemic, a set of interconnected and overlapping outcomes (Unick et al. 2013; Valdiserri et al. 2014). A syndemic occurs when various conditions interact, amplifying the negative effects in a population (Singer et al. 2017). Specifically, Perlman and Jordan (2018) describe the opioid syndemic as being comprised of fatal and non-fatal overdose, opioid use disorder, rates of hepatitis C virus (HCV), and human immunodeficiency virus (HIV) infections. Opioid misuse is a primary driver of the opioid syndemic, but opioid misuse is challenging to observe directly at the population level. However, by jointly modeling the complex interactions between various opioid-related outcomes over space and time, we gain valuable insight and a better understanding of the common patterns of the opioid syndemic.

In recent years, North Carolina (NC) has experienced a significant increase in opioid-related death rates. In 2017, the death rate per 100,000 population in NC was 24.1, which exceeded the rates in neighboring states such as South Carolina and Virginia, but remained lower than Tennessee, Kentucky, and substantially lower than West Virginia. However, by 2021, the NC death rate had risen to 39.2 per 100,000, approaching the levels observed in Tennessee, Kentucky, and West Virginia (National Center for Health Statistics 2024). According to the NC Department of Health and Human Services (NCDHHS), the rate of emergency department (ED) visits in NC was 116 per 100,000 in 2018, which was lower than the national rate. However, by 2022, this rate had risen to 161.5 per 100,000, exceeding the nationwide rate (CDC 2024a; NCDHHS 2024). On the contrary, the rate of people living with HCV in North Carolina between 2013 and 2016 was lower than the rates observed in the southern region of the United States, as well as nationwide (HepVu 2024). This is likely due in part to the efforts of the NCDHHS to address HCV. These efforts include increased awareness among individuals at high risk, expanded HCV prevention education initiatives, improved vaccination coverage against hepatitis A and hepatitis B among high-risk populations, and enhanced education and linkage to care for HCV-infected individuals regarding liver health and treatment options (Rhea et al. 2016). Furthermore, the rate of people living with HIV in NC in 2021 is lower than in the Southern Region, but is similar to national rate (AIDSVu 2024). Examining how these outcomes interact jointly, as well as in meaningful pairs, allows us to study not only the overall burden of the opioid syndemic in NC, but also trends in overdose, treatment, and associated infectious diseases.

Spatial or spatio-temporal factor models can be used to jointly model multiple, intercorrelated outcomes. For instance, Wang and Wall (2003) developed a common spatial factor model to study multivariate spatial data on cancer risk across counties in Minnesota. Lopes et al. (2008, 2011) extend spatial factor models to the spatio-temporal setting, introducing a spatial dynamic factor model that captures spatial dependence through factor loadings and temporal dependence through latent factors. In contrast, Leorato and Mezzetti (2021) introduce a Bayesian hierarchical factor model that extends the dynamic latent factors to incorporate both spatial and temporal dependencies. Factor models assume that individual outcomes are driven by a set of shared latent variables, called factors. A general form for a spatio-temporal factor model at location $i$ and time $j$ is


(1.1) \begin{align*}\boldsymbol{Y}_{ij}=\boldsymbol{\Gamma}\boldsymbol{F}_{ij}+\boldsymbol{\epsilon}_{ij},\end{align*}

where $\boldsymbol{Y}_{ij}$ is the $p\times 1$ vector of observed outcomes, $\mathbf{F}_{ij}$ is a vector of $m\times 1$ vector of latent factors, $\boldsymbol{\Gamma}$ represents the $p\times m$ matrix of factor loadings, and $\boldsymbol{\epsilon}_{ij}$ represent the $p\times 1$ vector of independent and identically distributed random errors. The factors quantify variability that is shared among all or subsets of the outcomes.

A well-known challenge in factor analysis modeling is the identifiability of model parameters (Bollen 1989; Anderson 2003). This is visually apparent by looking at (1.1), which shows that an equivalent equation can be obtained if the loadings are right multiplied by an orthogonal matrix, provided that the factor is left multiplied by the transpose of that matrix. A standard solution to address this issue is to impose a suitable constraint on the loadings. This is often achieved by making the loadings matrix lower triangular and forcing the diagonal elements to be positive (Geweke and Zhou 1996; Aguilar and West 2000; Lopes and West 2004; Bai and Wang 2015), or, as in Frühwirth-Schnatter and Lopes (2024), by using generalized lower triangular matrices. A lower triangular constraint with ones as diagonal elements is adopted by Kline et al. (2023) in their study of the opioid syndemic in Ohio. The authors constructed three factors to quantify variation shared across multiple opioid-related outcomes. However, the lower triangular setup complicates interpretation of the resulting factors, because the lower triangularization of the loadings matrix constrains the grouping of the outcomes in ways that lack practical meaningfulness.

One specific type of factor model is confirmatory factor analysis (CFA) (Jöreskog 1969), where the researcher predefines the number of factors and the structure of the loadings. These models fall under a broader category of structural equation modeling (Bollen 1989; Sánchez et al. 2005), and a particular example within this class is the shared component model (Knorr-Held and Best 2001; Held et al. 2005; Richardson et al. 2006), commonly used to map two diseases and easily adaptable to the modeling of multiple outcomes. These models use unobserved factors to quantify variation shared by both diseases and those specific to each disease. The loadings matrix governs the connection between the outcomes (i.e. diseases) and the latent components. Relating these models to (1.1), one can think of the $\boldsymbol{\Gamma}$ matrix as having elements $\gamma_{ij}$, where each $\gamma_{ij}$ represents the loading (or weight) of the $j^{\mathrm{th}}$ latent factor on the $i^{\mathrm{th}}$ observed outcome. In practice, these $\gamma_{ij}$s are preassigned entries, with some set to zero and others to nonzero values, indicating the relationships between outcomes and factors. Extending shared component space-time models to incorporate more than two diseases (or opioid-related outcomes, as in our case) introduces challenges, particularly in formulating a unique model without encountering identification issues. This is due to the need for a loadings matrix setup that departs from the lower triangular structure to represent interactions among different subsets of diseases.

In our work, we propose a shared component/factor model for the spatio-temporal joint modeling of six outcomes reflective of the opioid syndemic. We specify the model in a way that estimates four spatio-temporal latent factors: one that quantifies variability shared by all outcomes and three that quantify remaining variability shared by pairs of outcomes. The resulting four factors are desirable from an interpretation standpoint, as the first can be interpreted as the overall severity of the opioid syndemic, and the latter three quantify remaining variability that is shared between the chosen pairs of outcomes. However, this formulation results in a non-lower triangular loadings matrix. We address the identification problem of our model by using a novel implementation consisting of matrix decomposition. The paper is organized as follows: Section 2 provides an overview of the data. In Section 3, we present our proposed modeling framework and describe our implementation strategy, including our approach to overcome the identification problem. Section 4 proves results of our analysis for the NC data, and the paper is concluded with Section 5, a discussion of our work.

## 2. NORTH CAROLINA OPIOID OUTCOME DATA

Our analysis includes various sources of surveillance data that are regularly monitored by NCDHHS. We define the opioid syndemic through the interaction of six outcomes: overdose deaths involving illicit opioids, ED visits related to drug overdose, treatment counts for opioid use disorder, counts of patients receiving a prescription for buprenorphine, total new cases of HCV (acute and chronic), and new diagnoses of HIV. The choice of these outcomes was driven by two factors: the belief of our subject matter collaborators that they effectively reflect various aspects of opioid misuse, and their public availability online. For each outcome of interest, we obtained yearly, county-level counts for each of NC’s 100 counties from 2017 to 2021 from the NC Opioid and Substance Use Action Plan (OSUAP) Data Dashboard (Fliss et al. 2023), the 2021 North Carolina Hepatitis B/C Surveillance report (North Carolina HIV/STD/Hepatitis Surveillance Unit 2021a), and the 2021 North Carolina HIV Surveillance Report (North Carolina HIV/STD/Hepatitis Surveillance Unit 2021b).

Specifically, overdose death counts include the number of unintentional, self-inflicted, assault, and undetermined overdose deaths involving illicit opioids, while ED visits include all ED visits related to drug and medication overdoses. Treatment admissions include the number of uninsured individuals and Medicaid beneficiaries with opioid use disorder served by treatment programs. State policy requires censoring of treatment counts between one and five, which we accounted for in the model. Buprenorphine counts are unique counts of residents who received a buprenorphine prescription. Both treatment admissions and buprenorphine serve as proxies for opioid use disorder. Detailed description of these variables and their original sources can be found in the Technical Notes section of the NC OSUAP Data Dashboard (Fliss et al. 2023). The HCV counts include cases of both acute and chronic hepatitis C, as defined by the Center for Disease Control and Prevention (CDC) (CDC 2024b), and HIV counts include the total number of newly diagnosed HIV infection cases. Furthermore, we obtain annual population estimates from the NC OSUAP data to calculate rates.

**Fig. 1.**
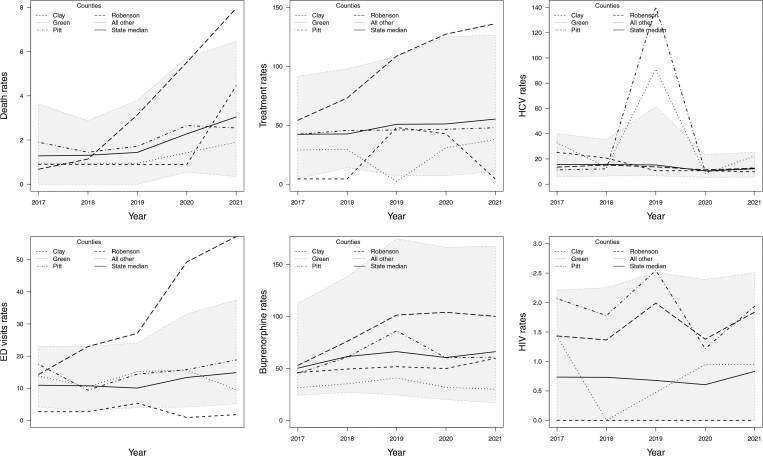
Rates per 10,000 residents of: death counts and ED visits (first column), treatment counts and buprenorphine treatments (second column), HCV infections and HIV infections (third column). The grey region represents all counties that fall within the $2.5$th quantile and $97.5$th quantile of the rates. The state median rate and specific counties are highlighted using different line types.

**Fig. 2.**
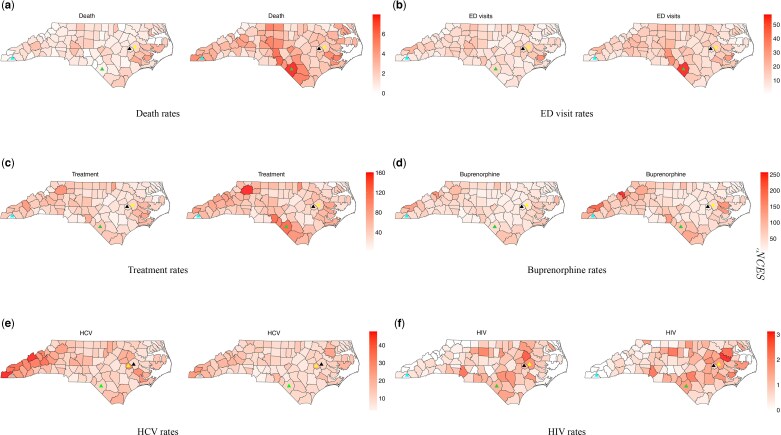
Rates per 10,000 residents of the six outcomes (a–f) in 2017 (first and third column) and 2021 (second and fourth column). The light blue triangle represents Clay county, black triangle represents Green county, yellow triangle represents Pitt county, and green triangle represents Robeson county.

Figure 1 shows time series plots of the rates per 10,000 of the six outcomes, revealing that the death rate and ED visits rate remained relatively flat from 2017 to 2019, but showed a significant increase in 2020. At the same time, treatment and buprenorphine prescription rate show a small but consistent increase over time. Furthermore, the HCV and HIV infection rates show considerable variability, as expected with small counts. Specifically, we observe a slight decrease in HCV infection rates over time, except for significant spikes in Pitt (with a population of size $180,742$ in 2021) and Green (with a population of size $20,524$ in 2021) counties in 2019. In contrast, HIV rates display substantial variability without a discernible temporal trend. Spatial snapshots of the rates per 10,000 residents of the six outcomes for 2017 and 2021 are shown in Fig. 2. Maps of the rates for all years are presented in Supplementary Figs S1–S6 in the Supplementary Material. In general, we observe an increase in treatment (Fig. 2c) and death (Fig. 2a) rates in the Southeastern Inner Coastal Plain and the Appalachian region of western NC. The prevalence of HCV (Fig. 2e) infection declines, while HIV (Fig. 2f) infection increases in some coastal counties of NC.

In Figs 1 and 2, we highlight several specific counties that exhibit noteworthy behavior. For example, we observe that Robeson County, with a population of size $130,625$ people in $2021$, has higher death rates, ED visits, and treatment rates compared to the state average, especially showing a significant increase from 2018. However, HCV infection rates are relatively similar to the state average, while HIV infection rates remain consistently above the state average, showing periods of increase and decrease during 2017–2021. In contrast, Clay County, with a population of $10,997$ people in 2021, has death rates below the state average until 2020, where a sharp increase is observed. ED visits remain significantly below the state average with a slight rise and subsequent decline. Treatment rates start at zero until 2018, then increase to reach the state average before decreasing again. HCV rates initially are above the state average in 2017 but decrease to align with the state average after 2019, while HIV rates remain constant at zero.

## 3. METHODOLOGY

In this study, we use a flexible approach that builds on spatial dynamic factor analysis. This methodology enables us to quantify variation shared among all six opioid-related outcomes or subsets of these outcomes through different spatio-temporal factors. Specifically, it provides flexibility to create factors that capture shared variation between all outcomes, in our case opioid overdose deaths, ED visits, treatment admissions, buprenorphine prescriptions, HCV, and HIV infections. Furthermore, our model framework includes factors that characterize interactions within pre-determined subsets of outcomes. In our analysis, the subsets of interest are the pairs deaths and ED visits, treatment admissions and buprenorphine prescriptions, and HCV and HIV infections. This approach allows us to quantify the overall shared variation and specific variation shared by meaningful subsets of outcomes, providing more detailed insights into the opioid syndemic.

### 3.1. Dynamic spatial factor modeling

Let $Y_{ijk}$ be the observed count of outcome $k$ in county $i$ and year $j$. In our work, we have $i = 1,\ldots,I = 100$ counties, $j = 1,\ldots,J = 5$ years ranging from 2017 to 2021, and $k = 1,\ldots, K = 6$ outcomes [death counts (D), ED visits (E), counts of people served by the treatment program (T), HCV infections (C), patients receiving buprenorphine (B), HIV infections (I)]. Let $\lambda_{ijk}$ be the relative risk for outcome $k$ in county $i$ and year $j$, relative to the baseline expected count of cases, $E_{ijk}$. That is, $E_{ijk}=P_{ij}r_{k}$, where $P_{ij}$ is the total population in county $i$ and year $j$ and $r_{k}$ is the overall state rate of the outcome $k$ in $2017$. We assume,


\begin{align*} Y_{ijk}\sim\mathrm{Poisson}(E_{ijk}\lambda_{ijk}),\end{align*}

where


(3.2) \begin{align*}\log(\lambda_{ijk})=\boldsymbol{\Gamma}_{k}^{^\prime}\mathbf{F}_{ij}+\epsilon_{ijk}.\end{align*}

In (3.2), $\mathbf{F}_{ij}$ denotes the $m$-dimensional vector of dynamic spatial factors in county $i$ and year $j$, $\boldsymbol{\Gamma}_{k}$ defines the $m$-dimensional vector of factor loadings for outcome $k$, and $\epsilon_{ijk}\overset{ind}{\sim}N(0,\sigma^{2}_{k})$ are error terms to account for uncorrelated heterogeneity and overdispersion. That is, $\epsilon_{ijk}$ is outcome specific variation that is not explained by the shared factors. Recall that treatment counts between 1 and 5 are censored and taken into account in the data model by adapting the censored generalized Poisson regression model of Famoye and Wang (2004).

We assume there are four factors (i.e. $m = 4$), where the first factor is shared by all outcomes. The remaining factors are shared only by pre-specified subsets of the outcomes in order to quantify remaining spatio-temporal heterogeneity that is common to outcomes in that particular subset after accounting for the variability that is shared by all outcomes. More specifically, the second factor is shared only by death and ED visits, the third factor is shared by individuals served by treatment programs and patients receiving buprenorphine, and the fourth factor is shared only by HCV and HIV infections. This specific pairing of the outcomes enables us to quantify various aspects of the opioid syndemic after accounting for the overall severity, with the second factor quantifying overdose, the third factor reflecting opioid use disorder, and the fourth factor related to infectious diseases. To comply with this structure, we assume that the $K\times m$-dimensional loadings matrix $\boldsymbol{\Gamma}$ takes the following form:


(3.3) \begin{align*}\boldsymbol{\Gamma}=\begin{bmatrix}\gamma_{D1}&\gamma_{D2}&0&0\nonumber\\\gamma_{E1}&\gamma_{E2}&0&0\nonumber\\\gamma_{T1}&0&\gamma_{T3}&0\nonumber\\\gamma_{C1}&0&0&\gamma_{C4}\nonumber\\\gamma_{B1}&0&\gamma_{B3}&0\nonumber\\\gamma_{I1}&0&0&\gamma_{I4}\end{bmatrix},\end{align*}

where $\gamma_{km}$ denotes the loading of factor $m$, $m = 1,\ldots,4$, for outcome $k\in\{D, E, T, C, B, I\}$, with the letters corresponding to the six outcomes as defined above.

To capture spatio-temporal autocorrelation in the latent factors, we assume an intrinsic conditional auto-regressive model (ICAR) (Besag et al. 1991) with an autoregressive of order 1 [AR(1)] (Box et al. 2015) temporal structure for each factor. Specifically, let $f_{ijm}$ denote the $m^{\mathrm{th}}$ element of $\mathbf{F}_{ij}$. We assume:


(3.4) \begin{align*} f_{i1m}|\mathbf{f}_{-i1m}&\sim N\left(\mu_{1m}+\sum\limits_{l}\frac{w_{il}}{w_{i+}}(f_{l1m}-\mu_{1m}),\frac{1}{w_{i+}}\right),\,\,\mathrm{for}\,\, j=1,\tag{3.4}\end{align*}

(3.5) \begin{align*} f_{ijm}|\{f_{i(j-1)m},\mathbf{f}_{-ijm}\}&\sim N\left(\tilde{\mu}_{ijm}+\sum\limits_{l}\frac{w_{il}}{w_{i+}}(f_{ljm}-\tilde{\mu}_{ljm}),\frac{1}{w_{i+}}\right),\,\,\mathrm{for}\,\, j=2,…,J,\tag{3.5}\end{align*}

where $\mathbf{f}_{-ijm}=\{f_{\ell jm}:\ell\neq i\}$ is the vector of factor $m$ for all counties excluding county $i$ in year $j$, $\mu_{jm}$ is the statewide mean of factor $m$ at year $j$, $\tilde{\mu}_{ijm}=\mu_{jm}+\eta_{m}(f_{i(j-1)m}-\mu_{(j-1)m})$, $\eta_{m}$ is a temporal autoregressive parameter for factor $m$, $w_{il}$ is an indicator of whether counties $i$ and $l$ are neighbors, and $w_{i+}$ is the total number of neighbors for county $i$. It is important to note that the conditional variance of the factors is fully specified, ensuring identifiability in the model. Specifically, this model structure yields a variance—covariance matrix of $\mathrm{Var}(\boldsymbol{F}_{ij}|\boldsymbol{F}_{i(j-1)})=\boldsymbol{I}_{m}$, where $\boldsymbol{I}_{m}$ is the $m\times m$-dimensional identify matrix, which is necessary to ensure identifiability (Bai and Wang 2015). Further details regarding model identifiability are in Section 3.2.

We use a Bayesian approach to model fitting, requiring the specification of prior distributions for all unknown parameters. We opt for weakly informative priors across all parameters. Specifically, $\eta_{m}\stackrel{{\scriptstyle indep.}}{{\sim}}\mathrm{Unif}(0,1)$ for $m = 1,\ldots,4$, $\sigma^{2}_{k}\stackrel{{\scriptstyle indep.}}{{\sim}}\mathrm{Inv-Gamma}(0.5,0.5)$ for $k = 1,\ldots,6$, and for each time-varying factor mean, $\mu_{jm}$ we assume independent uniform prior distribution on the real number line. Furthermore, we assume $\gamma_{D1}$, $\gamma_{E2}$, $\gamma_{T3}$, and $\gamma_{C4}$ are normally distributed with zero-mean and standard deviation of 10, but truncated to the positive real line to ensure at least one element in each column of $\boldsymbol{\Gamma}$ is positive, which is required for identifiability (Bai and Wang 2015). The remaining unconstrained $\boldsymbol{\gamma}_{km}$ are assumed to have zero-mean normal distributions with standard deviations of 10. To ensure the ICAR model yields a valid posterior distribution, we impose a required centering constraint for each latent factor and each year (Banerjee et al. 2014).

### 3.2. Model identification and estimation

Let $\log(\boldsymbol{\lambda}_{ij})=\left(\log(\lambda_{ij1}),\dots,\log(\lambda_{ijK})\right)^{^\prime}$ be the vector of the log relative risks for each outcome at location $i$ in year $j$. Then (3.2) implies


(3.6) \begin{align*}\mathbf{\log}(\boldsymbol{\lambda}_{ij})=\boldsymbol{\Gamma}\mathbf{F}_{ij}+\boldsymbol{\epsilon}_ {ij},\end{align*}

where $\boldsymbol{\Gamma}$ and $\mathbf{F}_{ij}$ are as defined above, and $\boldsymbol{\epsilon}_{ij}=(\epsilon_{ij1},…,\epsilon_{ijk})^{^\prime}$. The identifiability issue is made apparent by noting that the likelihood, $f(\mathbf{Y}_{ij}|\boldsymbol{\Gamma},\boldsymbol{F}_{ij},\boldsymbol{\epsilon}_{ij})$, is equivalent to $f(\mathbf{Y}_{ij}|\boldsymbol{\Gamma}\boldsymbol{H},\boldsymbol{H^{^\prime}F_{ij}},\boldsymbol{\epsilon}_{ij})$ for any $m\times m$ orthogonal $\boldsymbol{H}$. As previously mentioned, (3.6) can be made identifiable using a common triangularization constraint on the loadings matrix. Typically, in the literature, this constraint is used during the model setup, where the loading matrix is initially specified to be lower triangular. It is worth noting that in existing studies, estimation procedures may utilize this pre-defined triangular loadings matrix either during the pre-processing stage (Kaufeld et al. 2017; Leorato and Mezzetti 2021; Kline et al. 2023) or post-processing (Aßmann et al. 2016; Poworoznek et al. 2021). However, these approaches may result in factors lacking a meaningful interpretation. If the factor model framework is used solely for the purpose of dimension reduction, then this modeling choice is inconsequential. However, our goal is to derive factors that quantify meaningful relationships among subsets of outcomes that we a priori believe to be related, a key to understanding the opioid syndemic. Therefore, we deviate from the conventional lower triangular structure, as shown in (3.3), requiring a novel estimation method to ensure unique identification of the loadings and factors.

Specifically, we propose a solution using a $LQ$ decomposition (Ferguson et al. 1999) on the loadings matrix $\boldsymbol{\Gamma}$. That is, we seek unique matrices $\boldsymbol{L}$ and $\boldsymbol{Q}$ such that $\boldsymbol{\Gamma}=\boldsymbol{L}\boldsymbol{Q},$ where $\boldsymbol{L}$ is a lower triangular matrix with positive diagonal elements, and $\boldsymbol{Q}$ is an orthogonal matrix. Then, (3.6) becomes


(3.7) \begin{align*}\begin{array}{ll}\boldsymbol{\log(\lambda}_{ij})\hspace*{-4pt}&=\boldsymbol{L}\boldsymbol{Q}\mathbf{F}_{ij}+\boldsymbol{\epsilon}_{ij}\\&=\boldsymbol{L}\tilde{\mathbf{F}}_{ij}+\boldsymbol{\epsilon}_{ij},\end{array}\end{align*}

where $\tilde{\mathbf{F}}_{ij}\equiv\boldsymbol{Q}\mathbf{F}_{ij}$. With the LQ decomposition providing a lower triangular matrix $\boldsymbol{L}$ with positive elements on the diagonal and an orthogonal matrix $\boldsymbol{Q}$ that ensures $\mathrm{Var}(\tilde{\boldsymbol{F}}_{ij}|\tilde{\boldsymbol{F}}_{i(j-1)})=\boldsymbol{Q}\mathrm{Var}(\boldsymbol{F}_{ij}|\boldsymbol{F}_{i(j-1)})\boldsymbol{Q}^{^\prime}=\boldsymbol{I}$, both $\boldsymbol{L}$ and $\tilde{\boldsymbol{F}}$ become uniquely identifiable (Bai and Wang 2015).

To perform inference, the decomposition is enforced in the likelihood function. That is, we assume the likelihood depends on the loadings only through the decomposed matrices, i.e. $f(\boldsymbol{y}|\boldsymbol{\Gamma},\boldsymbol{F},\boldsymbol{\epsilon})=f(\boldsymbol{y}|\boldsymbol{L}(\boldsymbol{\Gamma}),\boldsymbol{Q}(\boldsymbol{\Gamma}),\boldsymbol{F},\boldsymbol{\epsilon})$, where the notation $\mathbf{L}(\boldsymbol{\Gamma})$, $\mathbf{Q}(\boldsymbol{\Gamma})$ is introduced to reinforce that the decomposed matrices are functions of the random matrix $\boldsymbol{\Gamma}$. This assumption ensures unique identifiability of the likelihood function. Others have used the LQ decomposition post-analysis to yield a lower triangular loadings matrix in order to ensure identifiability. However, we are not only interested in solving the identifiability issue, but also a primary goal of the analysis is estimation of the random matrices $\boldsymbol{\Gamma}$ and $\boldsymbol{F}$, as these model components were specified to provide meaningful interpretation of how the outcomes interact. Since these are parameters of interest for our analysis, we incorporate the decomposition into posterior calculations. Observe that our posterior distribution is then of the form


\begin{align*}\pi(\boldsymbol{\Gamma},\boldsymbol{F},\boldsymbol{\epsilon},\boldsymbol{\mu},\boldsymbol{\eta},\boldsymbol{\sigma}^{2}|\boldsymbol{y})\propto f(\boldsymbol{y}|\boldsymbol{L}(\boldsymbol{\Gamma}),\boldsymbol{Q}(\boldsymbol{\Gamma}),\boldsymbol{F},\boldsymbol{\epsilon})\pi(\boldsymbol{\Gamma})\pi(\boldsymbol{F}|\boldsymbol{\mu},\boldsymbol{\eta})\pi(\boldsymbol{\epsilon}|\boldsymbol{\sigma}^{2})\pi(\boldsymbol{\mu},\boldsymbol{\eta},\boldsymbol{\sigma}^{2}),\end{align*}

which is sampled using a Metropolis-within-Gibbs Markov chain Monte Carlo (MCMC) algorithm, implemented using the NIMBLE package (de Valpine et al. 2017) in R (R Core Team 2021).

For any given value of the loadings matrix, $\boldsymbol{\Gamma}$, the LQ decomposition is executed by first transposing the loadings matrix and separating it into a square full-rank matrix, $\boldsymbol{\Gamma}^{^\prime}_{1}$, and a rectangular matrix, $\boldsymbol{\Gamma}^{^\prime}_{2}$, as shown below:


(3.8) \begin{align*}\boldsymbol{\Gamma}^{^\prime}=\left[\begin{array}{c|c}\boldsymbol{\Gamma}^{^\prime}_{1}&\boldsymbol{\Gamma}^{^\prime}_{2}\end{array}\right]=\left[\begin{array} []{cccc|cc}\gamma_{D1}&\gamma_{E1}&\gamma_{T1}&\gamma_{C1}&\gamma_{B1}&\gamma_ {I1}\\\gamma_{D2}&\gamma_{E2}&0&0&0&0\\0&0&\gamma_{T3}&0&\gamma_{B3}&0\\0&0&0&\gamma_{C4}&0&\gamma_{I4}\end{array}\right].\end{align*}

We note that, in general, the setup of $\boldsymbol{\Gamma}$ in (3.3) may require reordering the outcomes to prevent a rank deficient square matrix $\boldsymbol{\Gamma}^{^\prime}_{1}$. The Gram-Schmidt process is used to compute the QR decomposition on $\boldsymbol{\Gamma}^{^\prime}_{1}$, yielding $\boldsymbol{\Gamma}_{1}^{^\prime}=\boldsymbol{Q}\boldsymbol{R}_{1}$, where $\boldsymbol{R}_{1}$ is an upper triangular matrix and $\boldsymbol{Q}$ an orthogonal matrix (Wong 1935). Define $\boldsymbol{R}=[\boldsymbol{R}_{1}|\boldsymbol{R}_{2}]$, where $\boldsymbol{R}_{2}=\boldsymbol{Q}^{^\prime}\boldsymbol{\Gamma}_{2}^{^\prime}$. Thus, we obtain $\boldsymbol{\Gamma}=\boldsymbol{LQ},$ where $\boldsymbol{L}=\boldsymbol{R}^{^\prime}$, and $\boldsymbol{Q}$ is the orthogonal matrix generated in the Gram-Schmidt process. In the MCMC algorithm, each random element of the loadings matrix is updated with a Metropolis-Hastings update. The above procedure for the LQ-decomposition is performed for every proposed value of $\boldsymbol{\Gamma}$. A single iteration of a Metropolis-Hastings update for $\boldsymbol{\Gamma}$ in the MCMC algorithm is depicted in Algorithm 1.

Algorithm 1
Metropolis-Hastings Update of Loadings

1. Let $\boldsymbol{\Gamma}$ denote the current value of the loadings matrix with $\boldsymbol{\Gamma}=\boldsymbol{L}\boldsymbol{Q}$.
2. Propose a new value $\gamma_{km}^{*}$ using a normal random walk, centered at the current value of $\gamma_{km}$. Let $\boldsymbol{\Gamma}^{*}$ be the loadings matrix containing the proposed $\gamma_{km}^{*}$.
3. Perform the LQ-decomposition of $\boldsymbol{\Gamma}^{*}$:

 Input$(\boldsymbol{\Gamma}^{*})$

– Let $\boldsymbol{\Gamma}^{*^{\prime}}=[\boldsymbol{\Gamma}^{*^{\prime}}_{1}\,\,|\,\,\mathbf {\Gamma}^{*^{\prime}}_{2}]$.
– Apply the QR Gram-Schmidt process to get $\boldsymbol{Q}^{*}$ and $\boldsymbol{R}_{1}^{*}$ such that $\boldsymbol{\Gamma}_{1}^{*^{\prime}}=\boldsymbol{Q}^{*}\boldsymbol{R}_{1}^{*}$.
– Compute $\boldsymbol{R}_{2}^{*}=\boldsymbol{Q}^{*^{\prime}}\cdot\boldsymbol{\Gamma}^{*^{\prime}}_{2}$.
– Let $\boldsymbol{R}^{*}=[\boldsymbol{R}_{1}^{*}\,\,|\,\,\boldsymbol{R}_{2}^{*}]$.
 **Output:** ($\boldsymbol{L}^{*}=\boldsymbol{R}^{*^{\prime}},\,\boldsymbol{Q}^{*}=\boldsymbol{Q}^{*}$)
  3. Compute the Metropolis-Hastings acceptance probability
  \begin{align*}\tau=\min\left(1,\frac{f(\boldsymbol{y}|\boldsymbol{L}(\boldsymbol{\Gamma}^{*}),\boldsymbol{Q}(\mathbf{\Gamma}^{*}),\boldsymbol{F},\boldsymbol{\epsilon})\pi(\boldsymbol{\Gamma}^{*})}{f(\boldsymbol{y}|\boldsymbol{L}(\boldsymbol{\Gamma}),\boldsymbol{Q}(\boldsymbol{\Gamma}),\boldsymbol{F},\boldsymbol{\epsilon})\pi(\mathbf{\Gamma})}\right).\end{align*}
4. Set $\boldsymbol{\Gamma}=\boldsymbol{\Gamma}^{*}$ with probability $\tau$.

## 4. RESULTS OF THE OPIOID SYNDEMIC IN NORTH CAROLINA

We implemented an MCMC algorithm for the model described in Section 3 using the NIMBLE package within R, for 500,000 iterations to fit the model. The initial 250,000 iterations are discarded as burn-in, and subsequently, every 50th sample is retained. Computation was done using a single core on the DEAC cluster (Information Systems and Wake Forest University 2021). Convergence was assessed by visually inspecting the trace plots. Note that the NIMBLE package did not include a built-in function for performing QR decomposition on a matrix, requiring that we develop a custom NIMBLE function to use within the package’s MCMC framework.

Spatial maps of the estimated log relative risk for each of the outcomes, displayed in Supplementary Figs S13–S18 in the Supplementary Material, closely resemble the maps of the empirical log relative risk displayed in Supplementary Figs S7–S12 in the Supplementary Material, reassuring the model’s goodness of fit to the data. Table 1 shows the posterior mean estimates of the factor loadings, $\boldsymbol{\Gamma}$, providing the necessary context to interpret the factor estimates. The first factor represents a synthesis of the six outcomes and is positively correlated with all outcomes except HIV, with which is negatively correlated. We note that death counts, treatment counts, and buprenorphine prescriptions receive similar weights, suggesting that these outcomes are more closely related to each other within the context of the opioid epidemic than to ED visits, HCV, and HIV. This is reasonable since ED visits include all ED visits related to drug and medication overdose, not solely opioid misuse, and HCV and HIV can also be acquired through other modes of transmission. Factor 2 is shared by death counts and ED visits, with death counts receiving a larger estimated loading than ED visits. Factor 3 combines the counts of people served by the treatment program and patients receiving buprenorphine, and both outcomes have similar loadings. Finally, Factor 4 intertwines HCV and HIV counts, with HIV counts bearing a smaller loading.

**Table 1.** Posterior mean estimates and $95\%$ credible intervals for the factor loadings on each outcome for each of the four factors.

Outcome	Factor 1	Factor 2	Factor 3	Factor 4
Death	0.53 (0.43, 0.63)	0.55 (0.46, 0.64)	0	0
ED visits	0.26 (0.18, 0.35)	0.35 (0.29, 0.42)	0	0
Treatment	0.67 (0.54, 0.76)	0	0.39 (0.28, 0.53)	0
HCV	0.43 (0.36, 0.50)	0	0	0.30 (0.23, 0.38)
Buprenorphine	0.68 (0.57, 0.76)	0	0.29 (0.22, 0.42)	0
HIV	–0.36 (–0.47, 0.23)	0	0	0.13 (0.05, 0.24)

HCV indicates hepatitis C virus; HIV, human immunodeficiency virus.

**Fig. 3.**
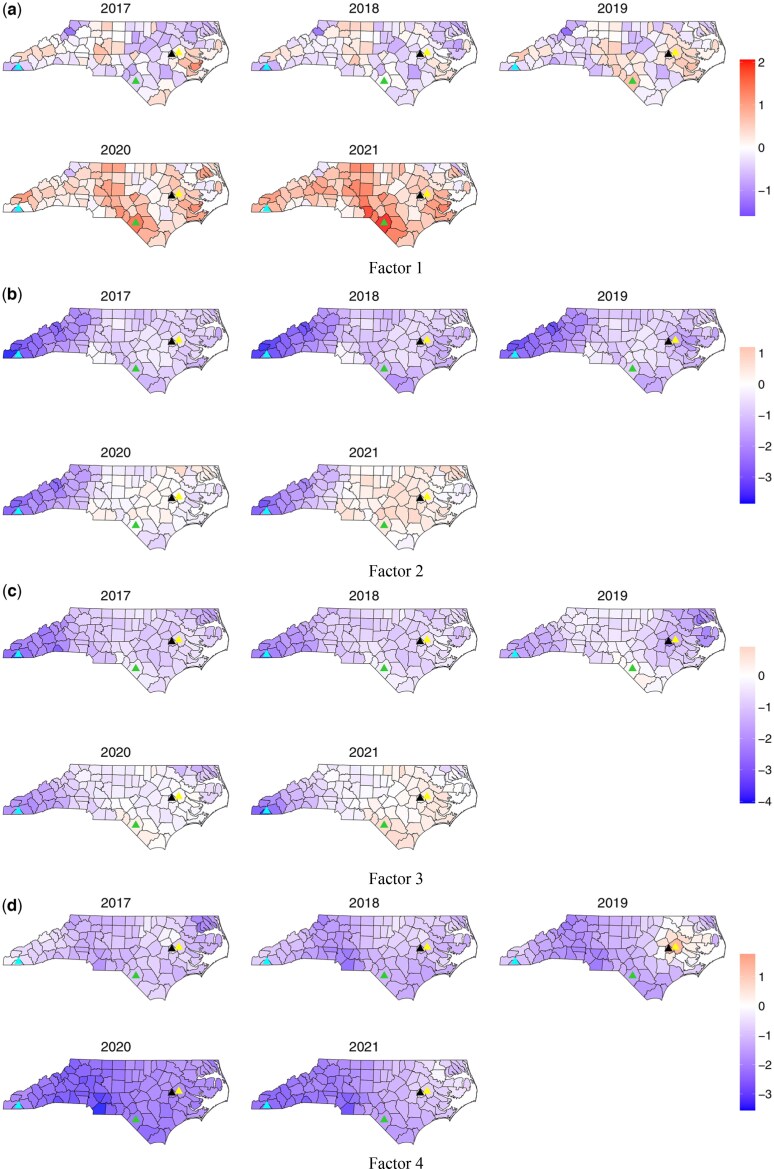
Posterior mean estimates of the four factors (a–d, respectively) from 2017 to 2021. The light blue triangle represents Clay county, black triangle represents Green county, yellow triangle represents Pitt county, and green triangle represents Robeson county.

**Fig. 4.**
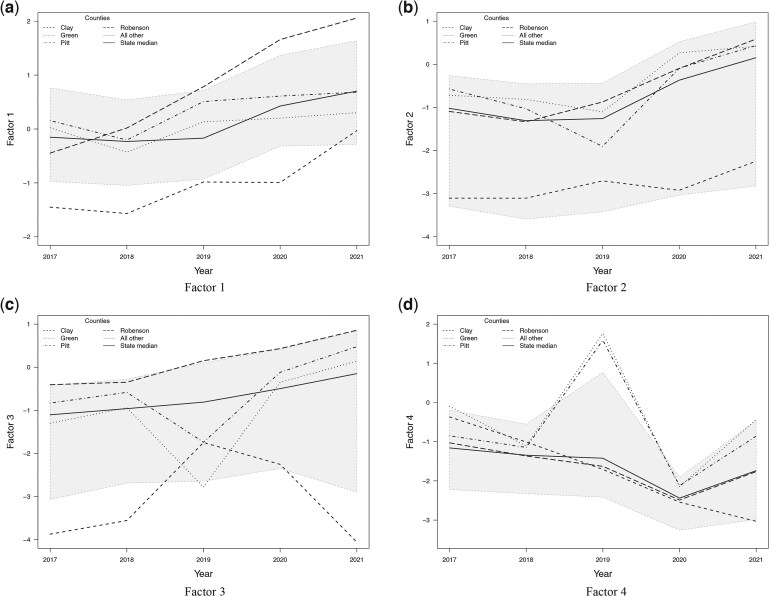
Posterior mean estimates of the four latent factors over time (a-d respectively). The grey region represents all counties that fall within the $2.5$th quantile and $97.5$th quantile of the rates. The state median and specific counties are highlighted using different line types.

Our interest is to understand how the interaction of all outcomes and subsets of all outcomes varies over space and time to learn about the opioid syndemic in NC. The posterior mean estimates for the four factors are depicted in Fig. 3a–d, and recall that these factors quantify variability that is shared in the log relative risk, relative to the state average for each outcome in 2017. In our analysis, we highlighted several specific counties where the factor estimates demonstrate noteworthy behavior. Figure 3a displays the posterior mean of Factor 1, while spatial maps depicting the standard deviation and residual for this factor are provided in Supplementary Figs S19 and S29–S34 in the Supplementary Material, respectively. As mentioned above, this factor captures the variation common to all six outcomes, and is strongly correlated with death counts, treatment counts, and buprenorphine prescription counts. The estimate of Factor 1 can be viewed as a latent burden of the syndemic (Kline et al. 2023) over space and time that underlines the shared variation of all outcomes. Overall, Factor 1 is showing an increasing trend throughout the state, with a more evident increase in the southern Piedmont Region. Figure 4a shows a time series of the posterior estimate of Factor 1 for each county along with the state average that aligns with Fig. 3a. Our observations reveal an increasing trend in Robeson County after 2017, exceeding the state average from 2018 onward. This result is consistent with the data for this county, where death, treatment, and buprenorphine prescription rates exhibited a substantial increase since 2018. On the contrary, Clay County, while below the state average, shows a significant increase after 2018. Once again, this result is consistent with the data, as we recall that death rates exhibited a significant increase starting in 2020, while treatment and buprenorphine prescription rates remained below the state average.

The posterior means of Factor 2 are shown in Fig. 3b, with the corresponding map illustrating the standard deviation for this factor included in Supplementary Fig. S20 in the Supplementary Material. Factor 2 quantifies the shared variation in the illicit opioid overdose death counts and drug overdose related emergency department visit counts, after Factor 1. That is, Factor 2 quantifies overdose and highlights counties where death and ED visit counts deviate from their expected value based on the shared variation once the variation across all outcomes is considered. The mean temporal trend of Factor 2 illustrated in Fig. 4b indicates a mostly negative but increasing trend over time. The increasing trend is also evident in the spatial distribution of Factor 2, with a higher rate in the Piedmont and Coastal Plain regions, but a lower rate in the Appalachian Region of Western NC. This factor highlights an essential aspect of factor analysis: Factor 2 explains the residual variation after the previous factor accounted for the shared variation among all outcomes. In particular, Factor 2 exhibits a spatial and temporal trend contrary to expectations; despite the anticipated increase in deaths and ED visits over time with the worsening of the opioid epidemic in NC, an inspection of the spatial plots of this factor reveals negative values in some of the NC regions and just slightly positive values in others. However, the temporal trend suggests an overall increase. It is essential to recognize that Factor 2 addresses the shared variation by death counts and ED visits after Factor 1 has explained the shared variation between all outcomes. Thus, Factor 2 aims to adjust any potential overrepresentation by death counts and ED visits in Factor 1, all while adhering to the increasing trend. Both spatial maps and time graphs reveal positive values mainly in 2020–2021, suggesting a correlation with high rates of synthetic opioid-related incidents, such as fentanyl (Kekatos 2023). This implies that in later years certain regions experienced increased overdose rates relative to the overall burden already captured by Factor 1. In the time series of this factor, it is evident that Robeson County has an increasing pattern above the state average, which is expected given the observed rise in the observed death rates and ED visits. Clay County, however, has a Factor 2 considerably below the state average, however, with an overall increasing trend observed in death counts and ED visits in the data.

In Figs 3c and 4c, we present the posterior mean of Factor 3 and the time series of the averages of the posterior mean estimates, respectively. The spatial maps depicting the standard deviation for this factor are provided in Supplementary Fig. S21 in the Supplementary Material. Factor 3 captures the shared variation between treatment and buprenorphine counts that has not been explained by Factor 1. That is, Factor 3 quantifies the treatment aspect of the opioid syndemic and highlights counties where treatment and buprenorphine prescriptions deviate from their expected value based on the shared variation once the variation across all outcomes is considered. This factor exhibits an increasing but overall negative trend both spatially and temporally, with lower estimates in the Appalachian Region of Western NC. Our time series analysis indicates that Factor 3 is above the state average for Robeson County with a significant increase after 2018. This pattern closely reflects the observed treatment counts and buprenorphine prescriptions. Specifically, there is a minimal change in this factor between 2017 and 2018, followed by a significant increase. This pattern is in alignment with Factor 2, which indicates a decrease in the shared variation of death counts and ED visits until 2018 and then a significant increase. In the case of Clay County, this factor is below the state average, following a pattern similar to the observed treatment counts. This consistency is expected, considering treatment counts have higher loadings for this particular factor. Moreover, it is interesting that Factor 3 for Green County follows a pattern similar to the observed treatment counts rather than buprenorphine prescriptions. Specifically, it shows a dip in 2019, followed by a significant increase in 2020. This result aligns with the higher weight placed on treatment counts than buprenorphine within this factor.

Figures 3d and 4d depict the posterior mean of the estimates of Factor 4 and the time series of the averages of the posterior mean estimates, respectively. The spatial maps depicting the standard deviation for this factor are provided in Supplementary Fig. S22 in the Supplementary Material. This reflects the remaining shared variation between HCV and HIV that is not already accounted for by Factor 1, and it serves as a means to understand the infectious disease elements of the opioid syndemic. The spatial and temporal trend of this factor show an overall decrease for this factor particularly evident in the Appalachian Region and Western Piedmont areas. However, the Coastal Plain Region of NC (e.g. Green and Pitts counties) displays a distinct pattern, with a significant increase observed in 2019, followed by a decrease in 2020 and a slight increase in 2021. For Robeson County, this factor is below or at the state average, aligning with the observed pattern in HCV infections data rather than following the trend seen in HIV infections data. This alignment is expected given the higher loadings of HCV infections in this factor. For Clay County, this factor shows a constant decrease that aligns with the pattern observed in the HCV infections data. This is in line with our expectations, considering that HIV infections are zero in this county.

We provide spatial plots of the posterior mean of the estimated uncorrelated heterogeneity for HCV (i.e. $\epsilon_{ijC}$) in Supplementary Fig. S23–S28 in the Supplementary Material. An interesting finding from these spatial plots pertaining to HCV indicates a high residual value for Pitt County. This observation aligns with what is already known that the HCV outbreak may not be related to injection-based transmission, but rather to increased screening efforts following the assignment of the new bridge counselor to cover the northeastern part of North Carolina (North Carolina HIV/STD/Hepatitis Surveillance Unit 2021a). Furthermore, spatial plots of the posterior mean of the estimated uncorrelated heterogeneity for HIV (i.e. $\epsilon_{ijI}$) indicate higher residual values in urban areas. This suggests that HIV transmission may also not be associated with injection-based transmission, but rather to other methods of transmission. Finally, in Supplementary Table S1 in the Supplementary Material we show the proportion of the variability in each of the six outcomes explained by the factors. It indicates that the majority of the variability in each outcome is accounted for by these factors.

Together, the four factors provide us with a meaningful interpretation of the various aspects of the opioid syndemic in North Carolina, highlighting increases in burden and overdose aspects between 2017 and 2020. They also allow us to identify regions with a higher demand for treatment services and shed light on the diseases-related aspect of the syndemic. Moreover, the model effectively captures extreme values by distinguishing whether these extremes are related to the opioid epidemic or other factors as we seen in the case of Pitt county. In 2019, the high Factor 4 values for this county suggest that some of the extreme values observed in the data are shared within HCV and HIV infections, while the residual plot indicates that our model can also differentiate between extremes influenced by factors unrelated to the opioid epidemic. The robustness and accuracy of the model, even with extreme values, are supported by the spatial maps of the posterior mean estimated log standardized relative risk, provided in the Supplementary Material, which align closely with the empirical log relative risk maps.

## 5. DISCUSSION

In this study, we develop a Bayesian hierarchical spatial dynamic factor model that describes multiple components of the opioid syndemic in NC. Such models allow the formulation of various combinations of outcomes to derive meaningful factors that help explain the intricate relationship between different outcomes. However, such typical combinations can lead to unidentifiable factor modeling. We address this issue by using the LQ decomposition algorithm to break down the unidentifiable loadings matrix. The decomposition yields a lower triangular matrix $\boldsymbol{L}$ with positive elements in the diagonal and an orthogonal matrix $\boldsymbol{Q}$. Imposing the restriction of strictly positive diagonal elements in $\boldsymbol{L}$ results in a lower triangular matrix that conforms to the loadings constraints. Furthermore, assuming that there are uncorrelated factors with unit variance, this decomposition leads to new factors that are identifiable. Posterior inference is performed using the MCMC algorithm that combines the LQ decomposition algorithm with the spatial ICAR model and temporal AR(1) model.

We illustrate our method using yearly, county-level data from North Carolina, involving six different outcomes to model the syndemic of opioid misuse. We combine these outcomes to describe meaningful insights that help better understand the complexities of the syndemic, such as the burden of the syndemic (Factor 1), overdose related to opioids (Factor 2), treatment related to opioids (Factor 3) and the infectious disease components of the syndemic (Factor 4). Our model helps us identify regions with the highest overall burdens of opioid misuse, such as the Piedmont Region of NC. The counties in this region are predominantly rural, making them ideal settings for investigating opioid misuse and its broader effects on rural communities. Furthermore, we observe lower overdose and treatment levels in the Appalachian Region of Western North Carolina, as suggested by Factors 2 and 3. These factors provide an opportunity to investigate the availability and utilization of harm reduction services among individuals who misuse opioids in these communities. Factor 4 highlights counties where the infectious diseases components of the opioid syndemic are different from what would be expected, given the fact that we already accounted for the burden of the syndemic. For example, HIV transmission is largely sexual in NC at this time, but there is a potential risk associated with substance use if not adequately controlled (North Carolina HIV/STD/Hepatitis Surveillance Unit 2021b).

In comparing our method to alternative approaches that do not decompose the loadings matrix, such as the one presented in Kline et al. (2023), we find that while both approaches produce similar results in terms of overall fit, the factors generated by the method presented in Kline et al. (2023) rely on a lower triangular design of the loadings matrix, resulting in less meaningful factors in the context of the opioid syndemic. Their method produces a general factor shared by all outcomes, a second factor shared by treatment counts, HCV, and HIV infections, and a third factor shared by HCV, and HIV infections. Although these factors are mathematically sound, they do not provide the level of interpretability required to capture distinct characteristics of the opioid syndemic, such as the trends in treatment, overdose, and associated infectious diseases. By specifying how each factor influences particular outcomes, as we do in our approach, we provide a more meaningful understanding of the opioid syndemic.

We would like to highlight several critical aspects of the model. First, it is important to note that we did not use model selection tools to evaluate the loadings matrix. Our study uses CFA, where the structure of the loadings matrix is predetermined based on empirical or conceptual foundations, rather than being identified through model selection techniques. For instance, in our study, we assess the estimated factors by comparing the estimated log relative risk of each outcome with the empirical log relative risk or by examining the spatial maps of heterogeneity for each outcome to ensure they are consistent with the conceptual understanding of the outcomes. While our approach focuses on evaluating the model’s goodness of fit through comparisons between estimated and empirical spatial maps presented in the Supplementary Material, there may be settings where the appropriate groupings of loadings are less obvious. In such cases, Bayesian Information Criterion (BIC) as in Ghosh and Dunson (2009) or other model selection tools could be useful for determining the loadings matrix that best describes the data. However, this was not the approach taken in our current work.

Second, it is important to be aware of certain constraints in this approach. One such constraint is to avoid over-parameterization and ensure that the model provides a unique set of parameter estimates. Specifically, avoiding over-parameterization is essential to ensure that the model provides a unique set of parameter estimates. Over-parameterization can occur when the number of freely estimated parameters, such as loadings and variance components, exceeds the number of observed outcomes. As noted by Geweke and Zhou (1996), the observed outcomes mainly determine their mean and covariance structure, which limits the number of parameters that can be estimated. To avoid an underdetermined model, the number of outcomes $K$ should satisfy the condition $K \gt 2 m + 1$, where $m$ is the number of factors.

Finally, we designed our loadings matrix to construct four different factors that provide meaningful understanding of the opioid syndemic. This setup requires that factor one influences all outcomes, while the remaining factors affect only pairs of outcomes. In general, one can use alternative predefined setups, but they must address a crucial aspect: to ensure the effectiveness of our decomposition, $\boldsymbol{\Gamma}^{^\prime}_{1}$ in (8) must not be rank deficient. This can be achieved by requiring that each row of $\boldsymbol{\Gamma}^{^\prime}_{1}$ contains at least one nonzero element. Translating this requirement back to the loadings matrix $\boldsymbol{\Gamma}$, defined in (3), it means that the top $K\times K$, where $K = 4$ number of outcomes, must contain at least one nonzero loadings in each column. This condition may require permuting some of the original rows before applying our proposed method.

It is worth mentioning that if the primary interest lies in examining the temporal relationship between a specific outcome and other time-varying variables, distributed lag nonlinear models (DLNM) (Gasparrini et al. 2010) could be a useful approach. A hierarchical Bayesian distributed lag logistic regression model has been successfully applied in Gonsalves et al. (2023) to assess the temporal patterns of infectious disease and overdose rates in relation to HIV cases, demonstrating the effectiveness of DLNMs in public health research. In the context of our study, since it often takes time for some of our outcomes, such as HIV and HCV, to be detected, we could apply the principles of DLNMs by expressing these outcomes in terms of lagged factors. However, as DLNMs are primarily designed for time series analysis, they typically require more granular measurements than our yearly data provide. This would involve expressing the outcomes as a time series regression on factors from previous time points, adding complexity related to identifiability, which will be challenging to address but essential for ensuring the model’s unique solutions.

Our analysis has some limitations. Our model assumes constant loadings across space and time, meaning that the variation among the outcomes and also among the factors and each individual outcome, is constant in space and time. Given the dynamic nature of the opioid epidemic—with evolving support centers like Syringe Services Programs (SSP) and changing laws such as the Strengthen Opioid Misuse Prevention (“STOP”) Act—intervention programs across the state and over time can have diverse impacts on each of the six outcomes considered in our study. Consequently, assuming the same correlation between the outcomes and also between the factors and outcomes, might be considered somewhat simplistic. One could explore extensions using loadings that vary geographically and/or in time as in Harris et al. (2011) and Kline and Hepler (2021). Moreover, in our study, we incorporate temporal dependence by modeling the mean function of the conditional distribution of the factors using an AR(1) process, while spatial dependence is captured through the covariance using ICAR model covariance. This approach can be expanded by incorporating a non-separable spatio-temporal covariance function proposed by Cressie and Huang (1999) and Gneiting (2002).

The data used in this analysis are subject to several limitations that should be considered. First, overdose death counts are based on information reported in death certificates, which can contain inaccuracies in classifying deaths (Slavova et al. 2015). This could result in incomplete data for overdoses, where some cases may not be reported or could be misclassified. Second, many acute HCV cases could be asymptomatic, which can lead to missing data, as asymptomatic cases may not be identified or reported. Thirdly, the total count of HCV cases includes both acute and chronic cases, but the duration of chronic cases is unknown, which could impact the interpretation of trends over time. Finally, since HIV is primarily transmitted through sexual contact and the duration of infection is often unknown, analyzing these cases becomes more complicated. These limitations emphasize the importance of interpreting the results carefully and highlight the advantage of using a confirmatory factor analysis approach. Such an approach allows for the incorporation of multiple outcomes, which can help address each other’s limitations and capture different aspects of the syndemic that might be fully represented by fewer outcomes.

In summary, we developed a dynamic spatial factor model approach for the modeling of the opioid syndemic in North Carolina, specifically designed to explore the complex underlying drivers of the opioid syndemic. By estimating four latent factors, we can quantify spatio-temporal shared variation across multiple outcomes and gain insight into the relationships between outcomes within the syndemic. Our framework provides a practical method for synthesizing information and identifying important sources of variation to support future investigations and interventions addressing the impacts of the opioid syndemic.

## Supplementary Material

kxae052_Supplementary_Data

## Data Availability

The data and code for this analysis are available at https://github.com/evamurphy100/NCopioidSyndemic.
