# Supplementary material for: Understanding the opioid syndemic in North Carolina: A novel approach to modeling and identifying factors
Source: Biostatistics. 2025 Jan 27;26(1):kxae052. doi: 10.1093/biostatistics/kxae052 (PMC11823283; doi:10.1093/biostatistics/kxae052)
Supplement: kxae052_Supplementary_Data [file kxae052_supplementary_data.pdf]

Biostatistics (2024), **0**, 0, *pp.* 1–**??**
doi:10.1093/biostatistics/output

Supplementary material to Understanding the Opioid Syndemic in North Carolina: A Novel Approach to Modeling and Identifying Factors

EVA MURPHY*

Department of Statistical Sciences, College of Arts and Sciences, Wake Forest University, Winston-Salem, NC

DAVID KLINE, KATHLEEN L. EGAN, KATHRYN E. LANCASTER

Department of Biostatistics and Data Science, Division of Public Health Sciences, Wake Forest School of Medicine, Winston-Salem, NC

WILLIAM C. MILLER

Gillings School of Global Public Health, University of North Carolina Chapel Hill, Chapel Hill, NC

LANCE WALLER

Department of Biostatistics and Bioinformatics, Rollins School of Public Health, Emory University, Atlanta, GA

STACI A. HEPLER

Department of Statistical Sciences, College of Arts and Sciences, Wake Forest University, Winston-Salem, NC

In the following plots the **light blue** triangle represents Clay county, **black** triangle represents Green county, **yellow** triangle represents Pitt county, and **green** triangle represents Robeson county.

© The Author 2024. Published by Oxford University Press. All rights reserved. For permissions, please e-mail: journals.permissions@oup.com

SM 1. Data

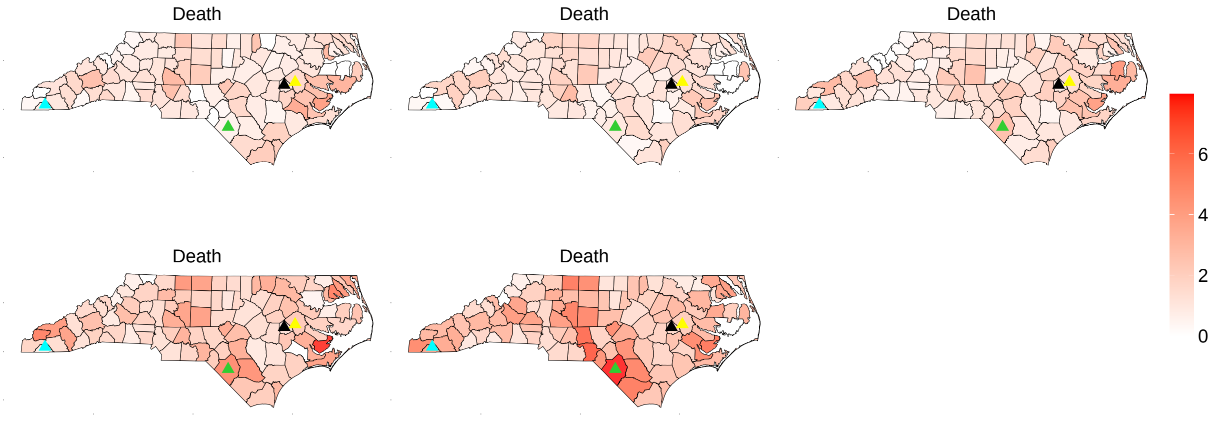

Fig. SM1. Death rates per 10000 resident from 2017 to 2021.

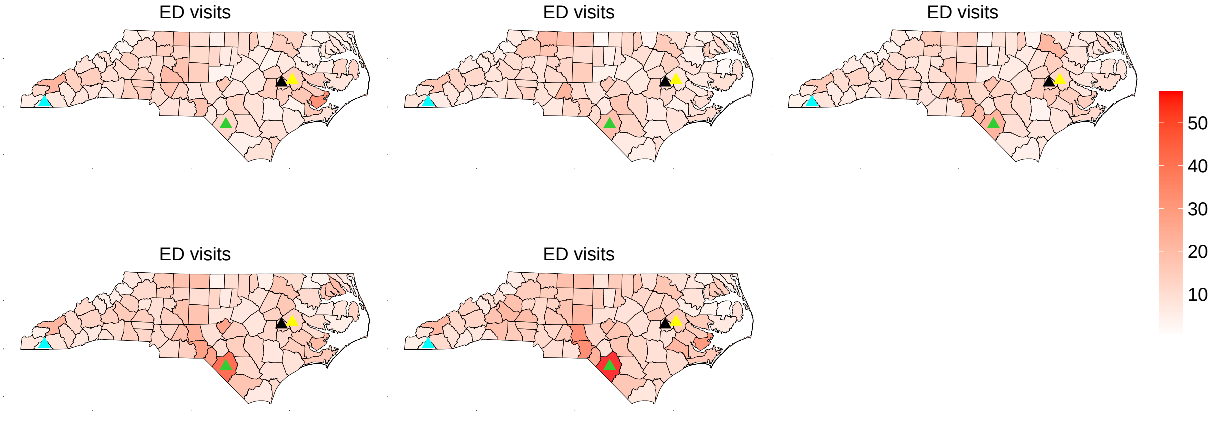

Fig. SM2. ED visit rates per 10000 resident from 2017 to 2021.

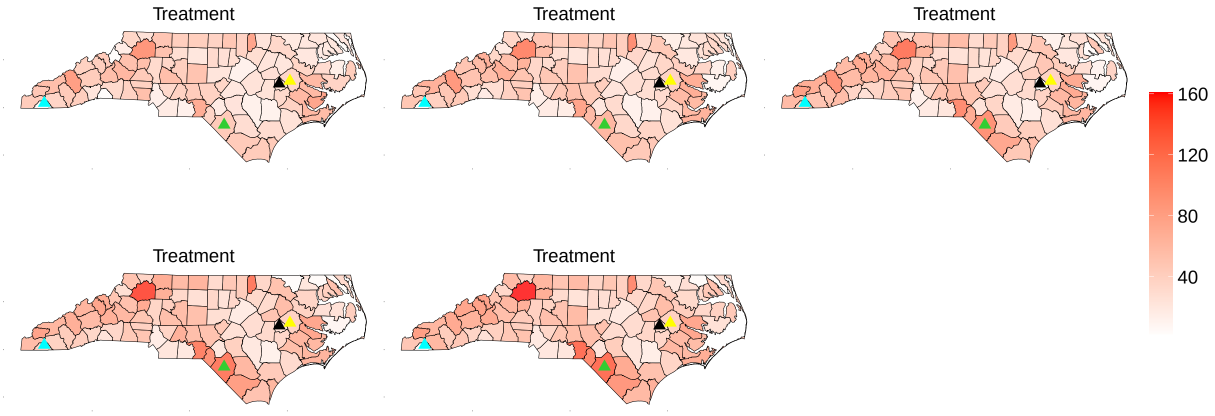

Fig. SM3. Treatment rates per 10000 resident from 2017 to 2021.

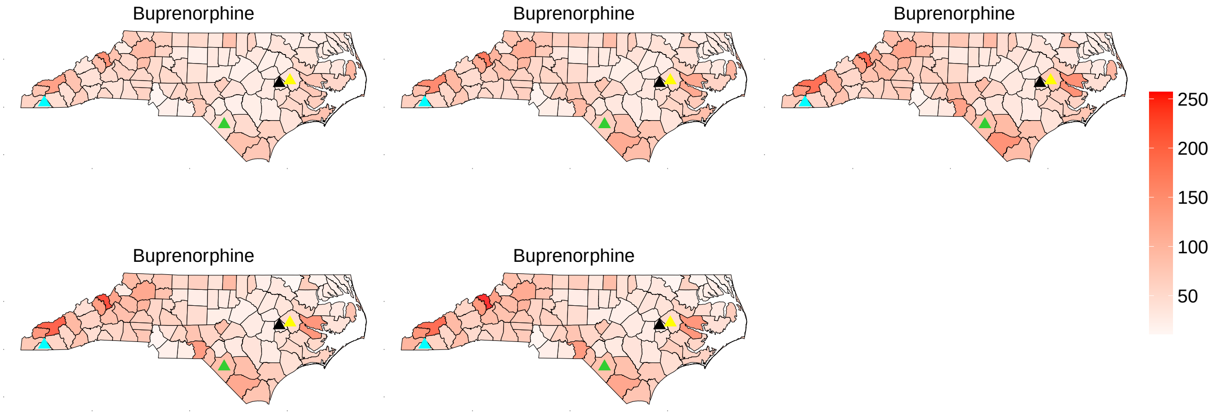

Fig. SM4. Buprenorphine prescription rates per 10000 from 2017 to 2021.

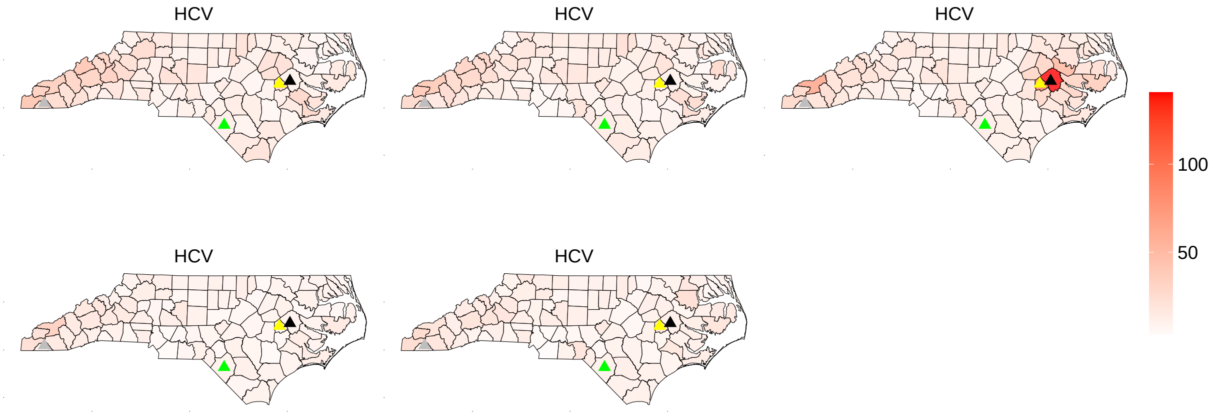

Fig. SM5. Total HCV infection rates per 10000 from 2017 to 2021.

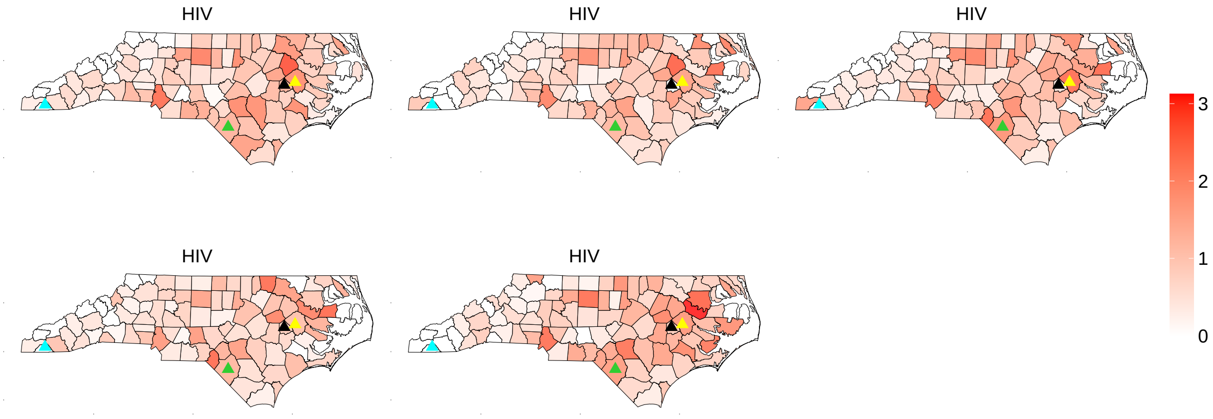

Fig. SM6. Newly diagnosed HIV infection rates per 10000 from 2017 to 2021.

SM 2. Observed log standardized relative risks

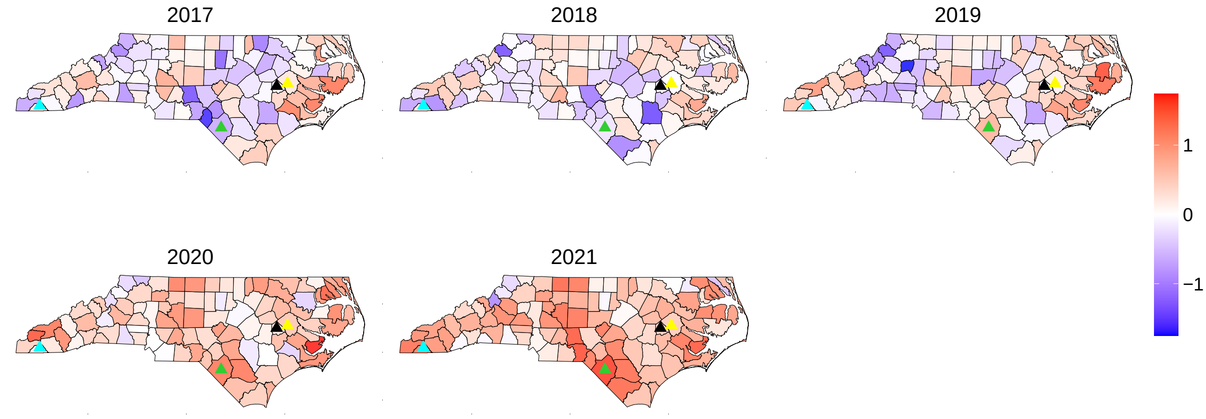

Fig. SM7. Observed log standardized relative risk for death counts from 2017 to 2021.

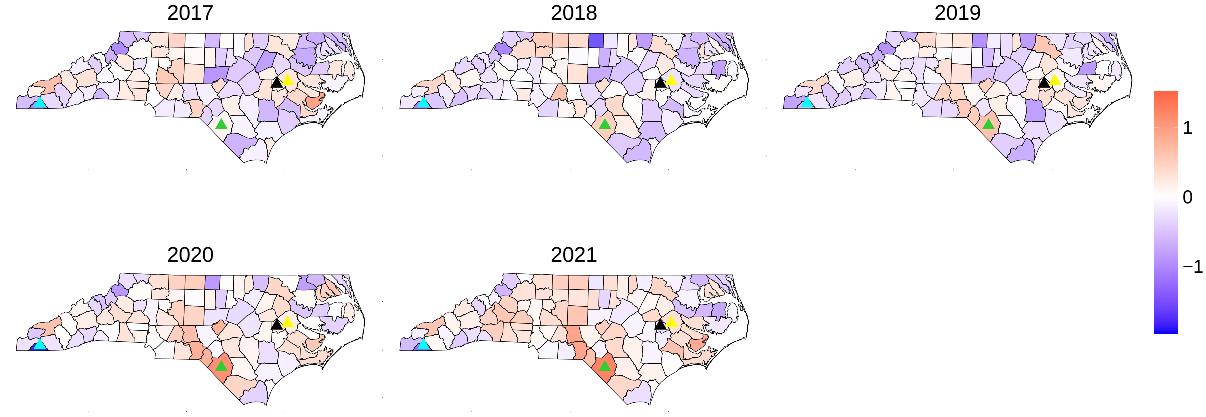

Fig. SM8. Observed log standardized relative risk for ED visits from 2017 to 2021.

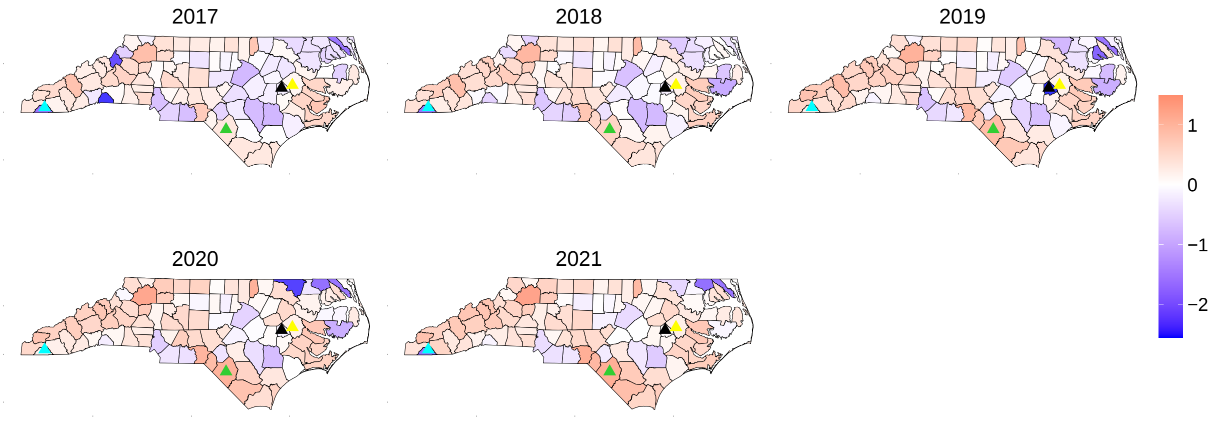

Fig. SM9. Observed log standardized relative risk for treatment counts from 2017 to 2021.

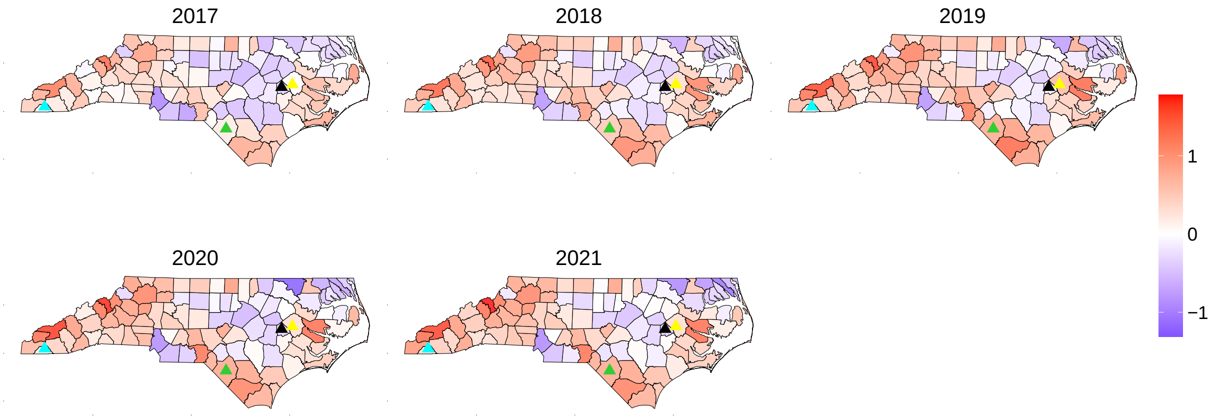

Fig. SM10. Observed log standardized relative risk for buprenorphine prescriptions counts from 2017 to 2021.

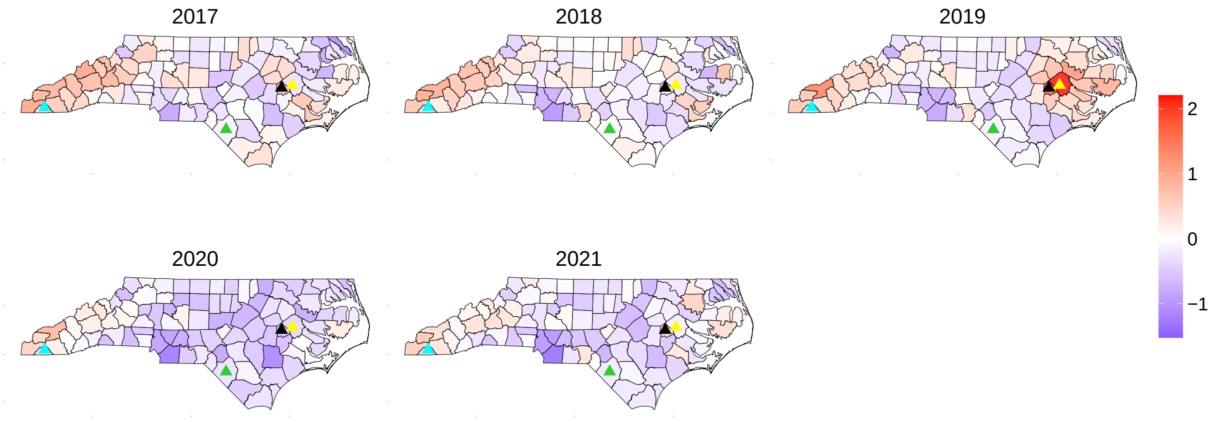

Fig. SM11. Observed log standardized relative risk for total HCV infections from 2017 to 2021.

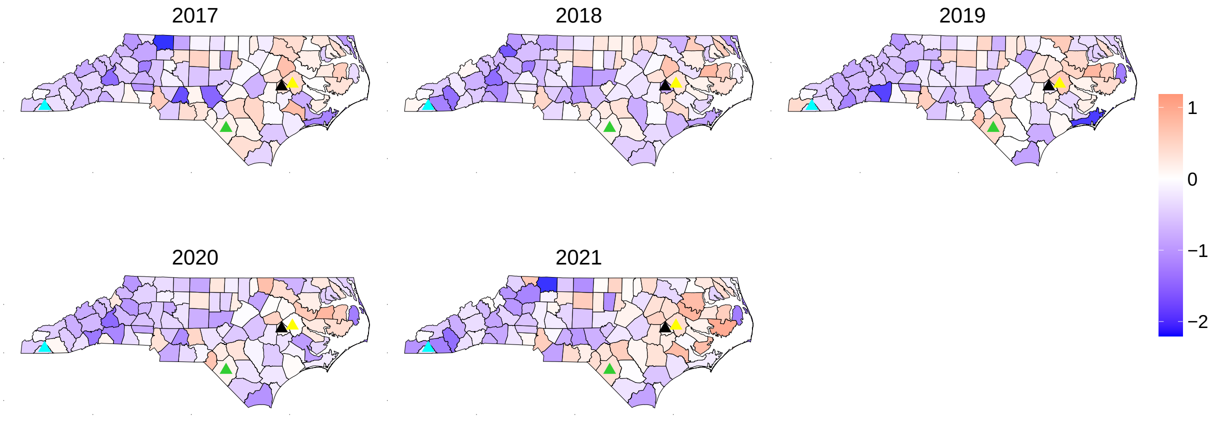

Fig. SM12. Observed log standardized relative risk for newly diagnosed HIV infections from 2017 to 2021.

SM 3. Posterior means of the estimated log standardized relative risk

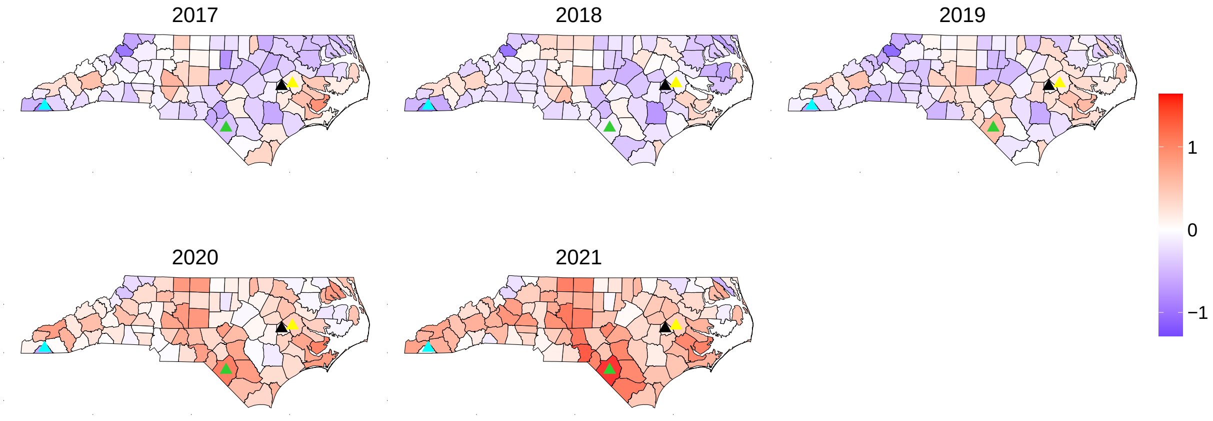

Fig. SM13. Posterior mean estimated log standardized relative risk for death counts from 2017 to 2021.

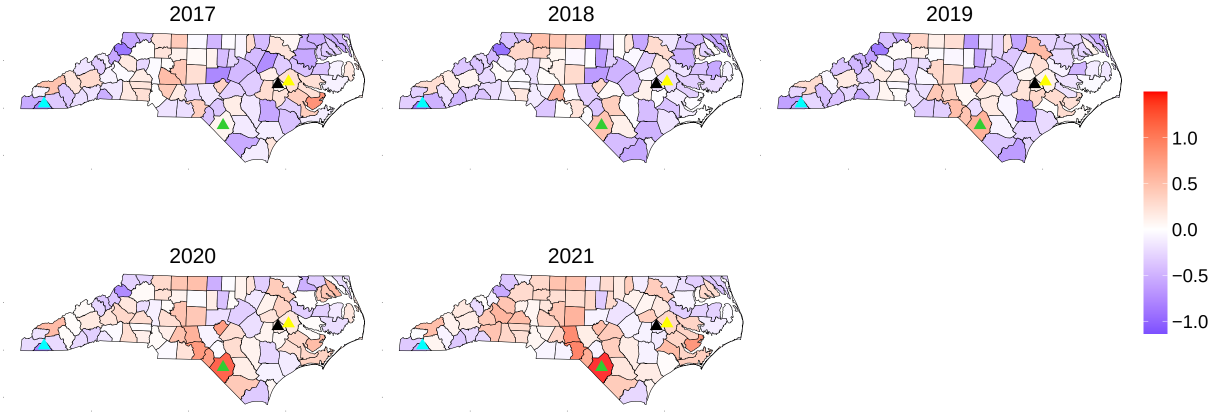

Fig. SM14. Posterior mean estimated log standardized relative risk for ED visits from 2017 to 2021.

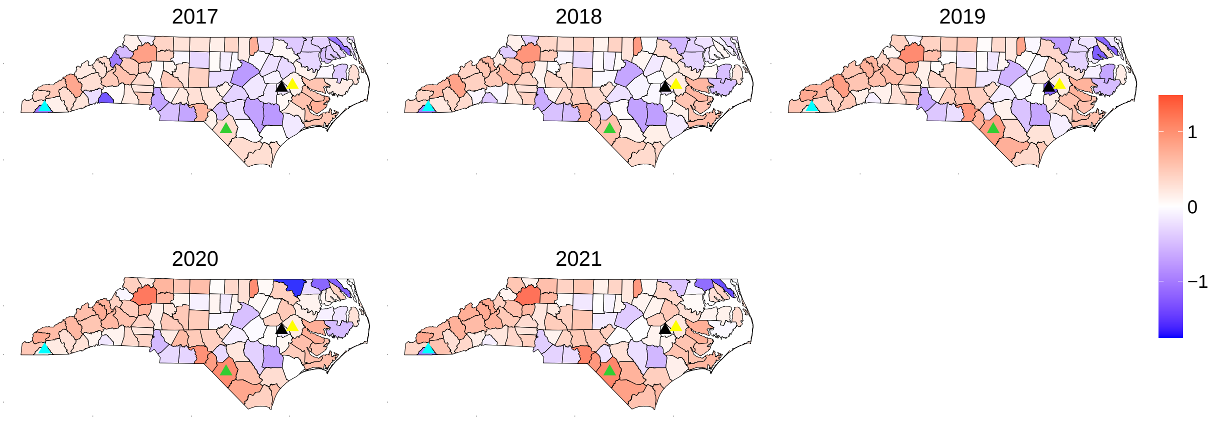

Fig. SM15. Posterior mean estimated log standardized relative risk for treatment counts from 2017 to 2021.

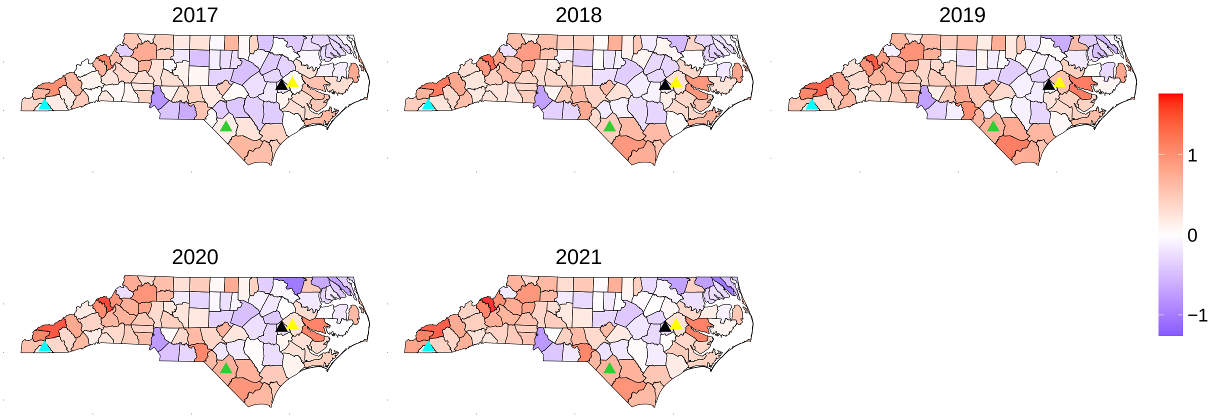

Fig. SM16. Posterior mean estimated log standardized relative risk for buprenorphine prescription counts from 2017 to 2021.

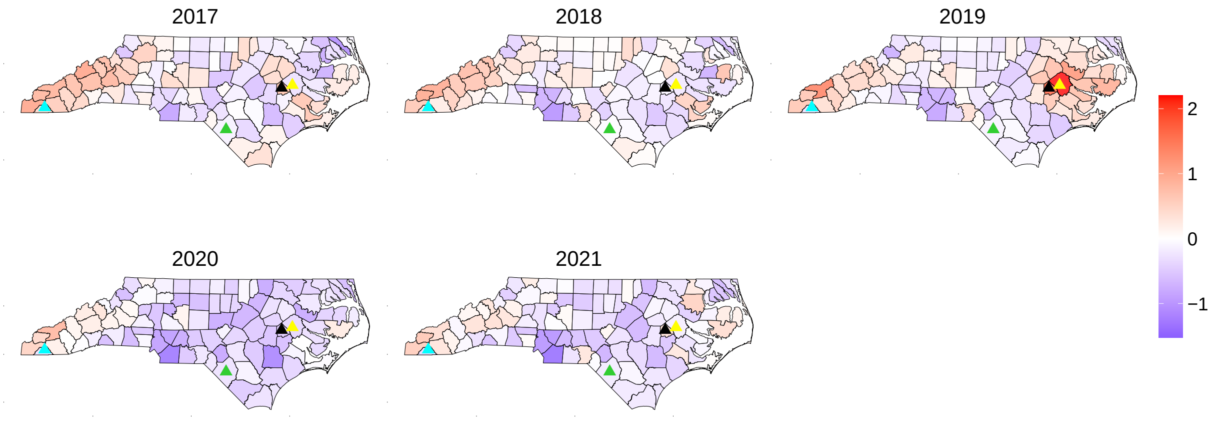

Fig. SM17. Posterior mean estimated log standardized relative risk for HCV infections from 2017 to 2021.

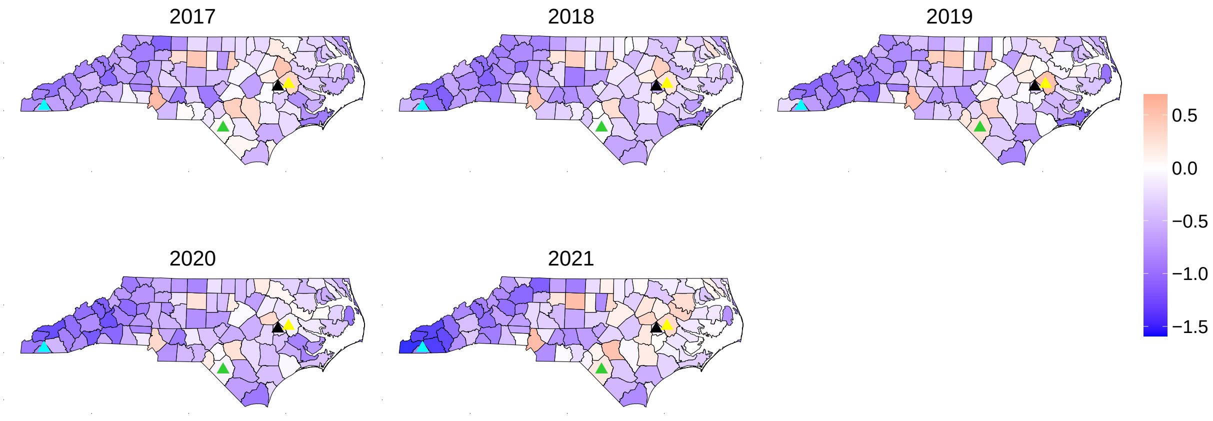

Fig. SM18. Posterior mean estimated log standardized relative risk for HIV infections from 2017 to 2021.

SM 4. Standard deviations of factor estimates

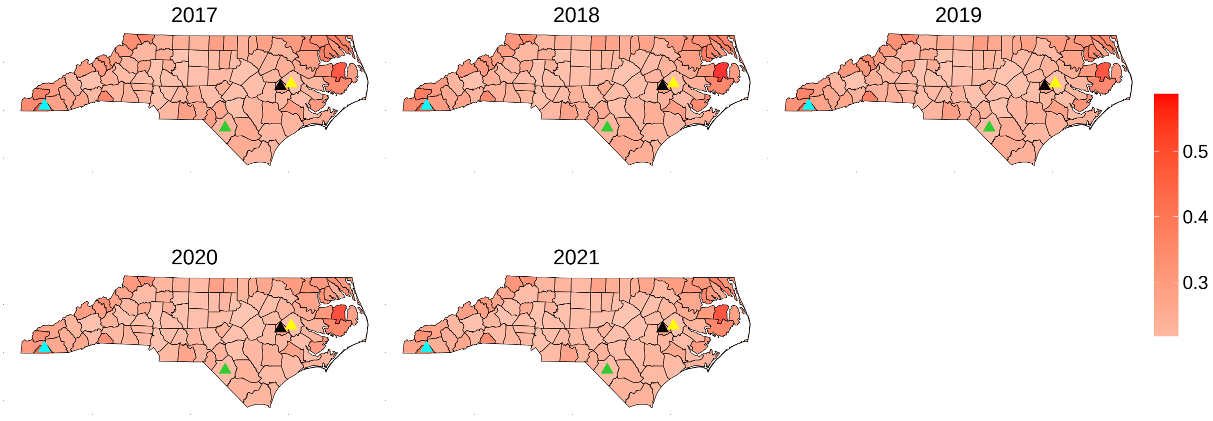

Fig. SM19. Standard deviation of factor 1 estimates

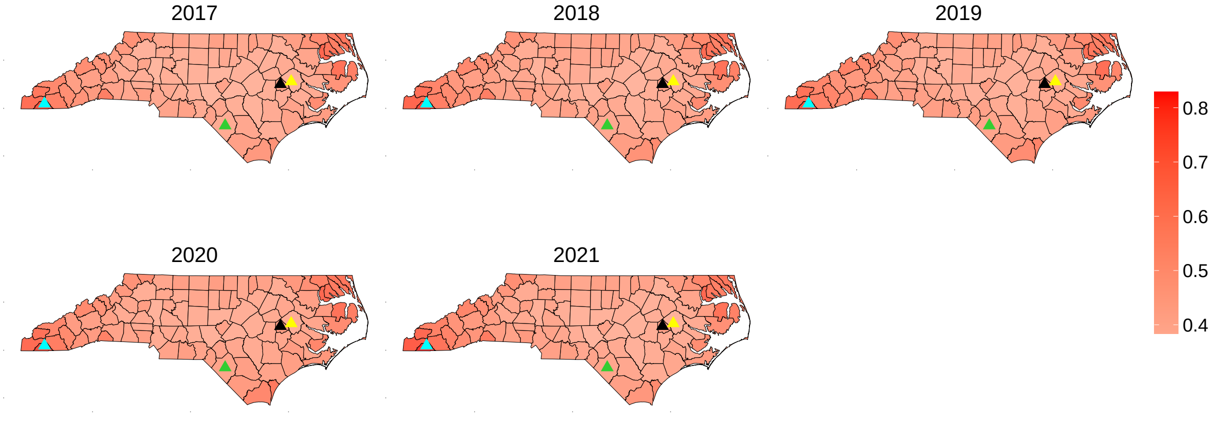

Fig. SM20. Standard deviation of factor 2 estimates

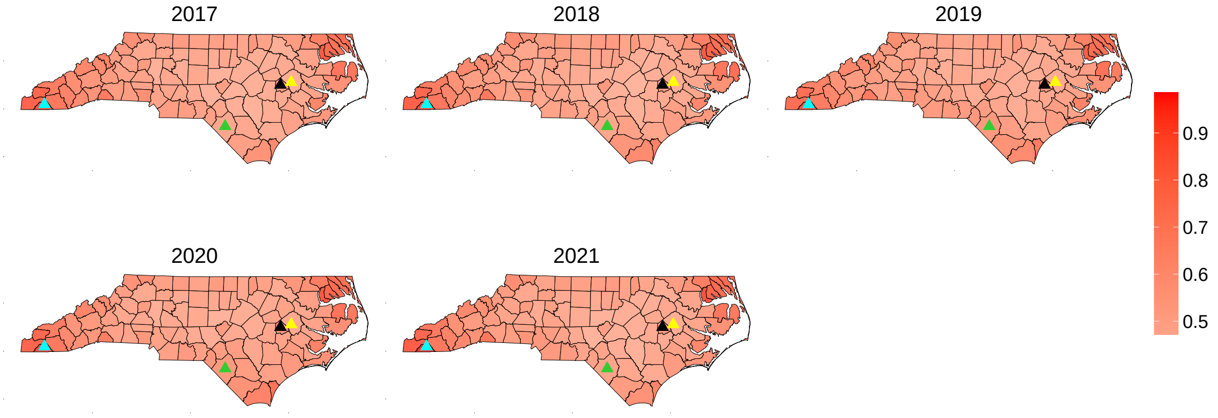

Fig. SM21. Standard deviation of factor 3 estimates

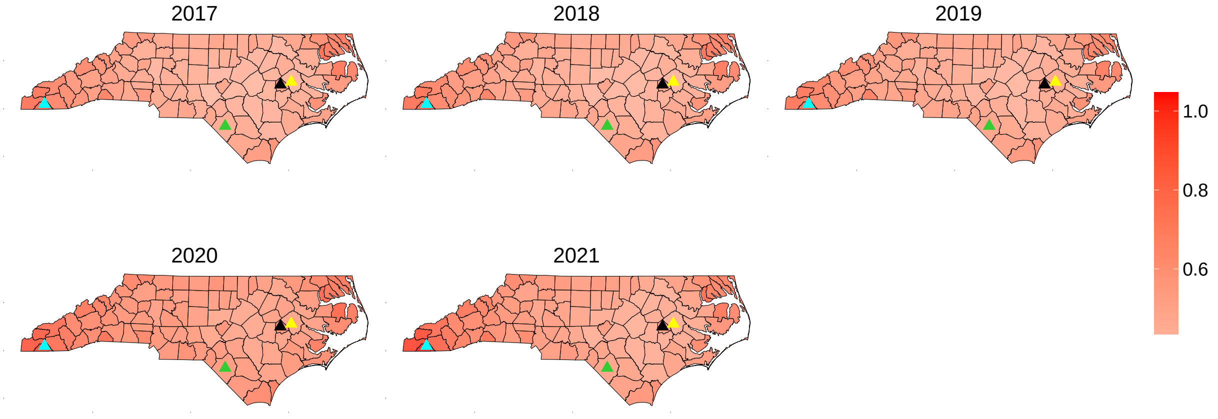

Fig. SM22. Standard deviation of factor 4 estimates

SM 5. Posterior mean of the estimated uncorrelated heterogeneity

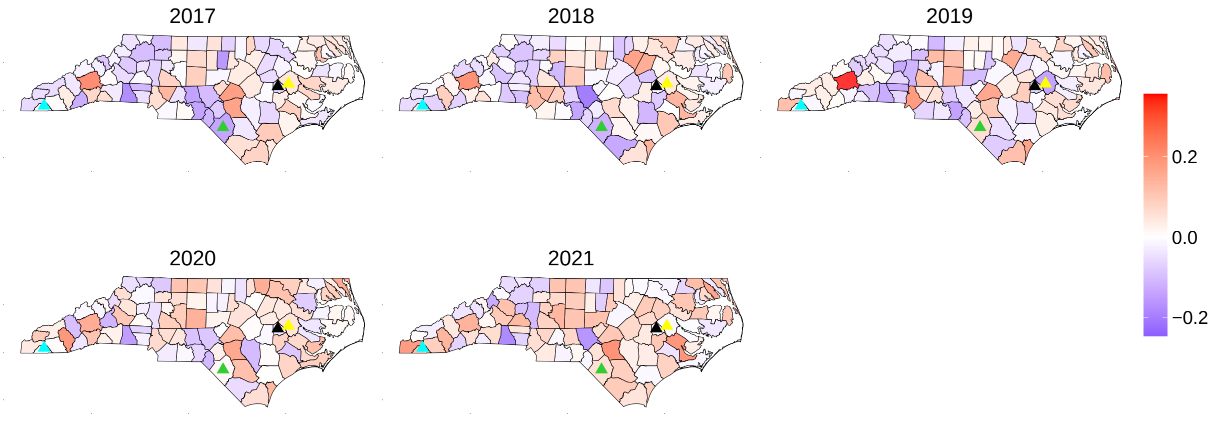

Fig. SM23. Posterior mean estimated uncorrelated heterogeneity for death counts from 2017 to 2021.

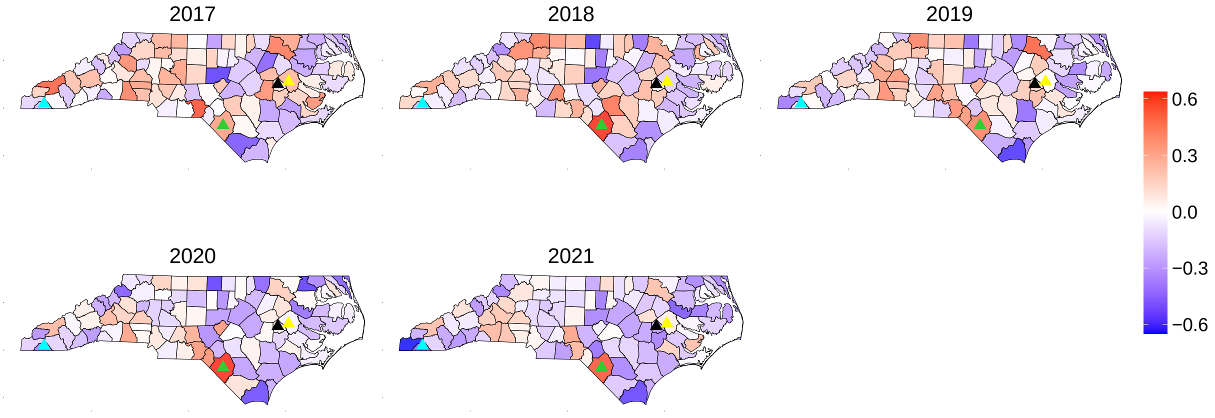

Fig. SM24. Posterior mean estimated uncorrelated heterogeneity for ED visits from 2017 to 2021.

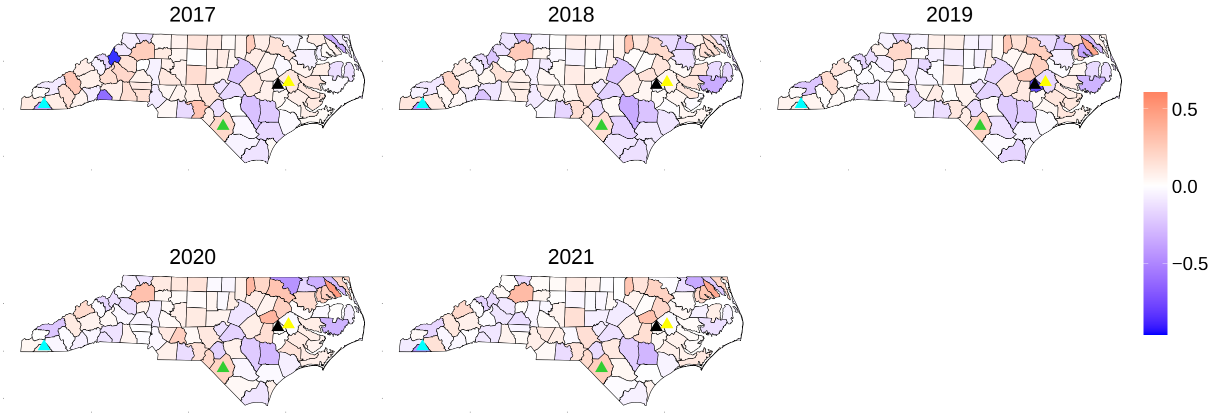

Fig. SM25. Posterior mean estimated uncorrelated heterogeneity for treatment counts from 2017 to 2021.

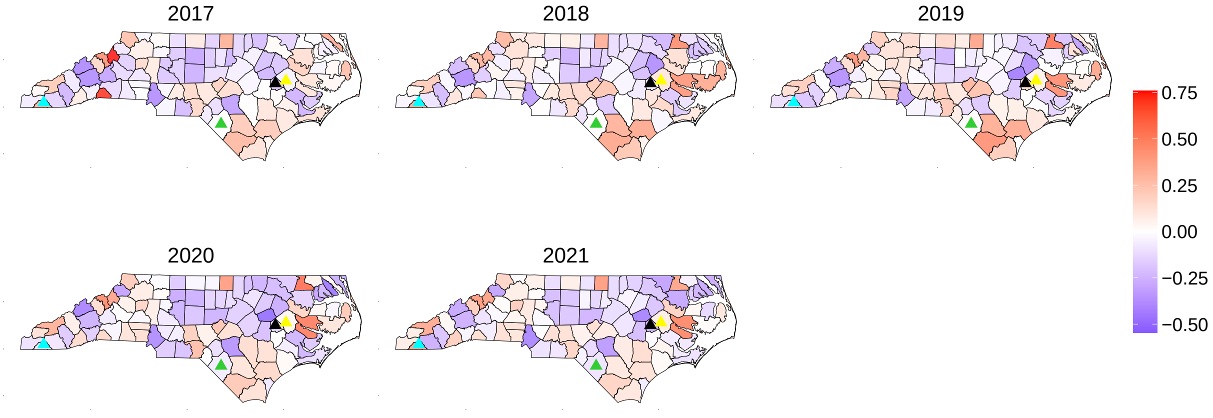

Fig. SM26. Posterior mean estimated uncorrelated heterogeneity for buprenorphine prescription counts from 2017 to 2021.

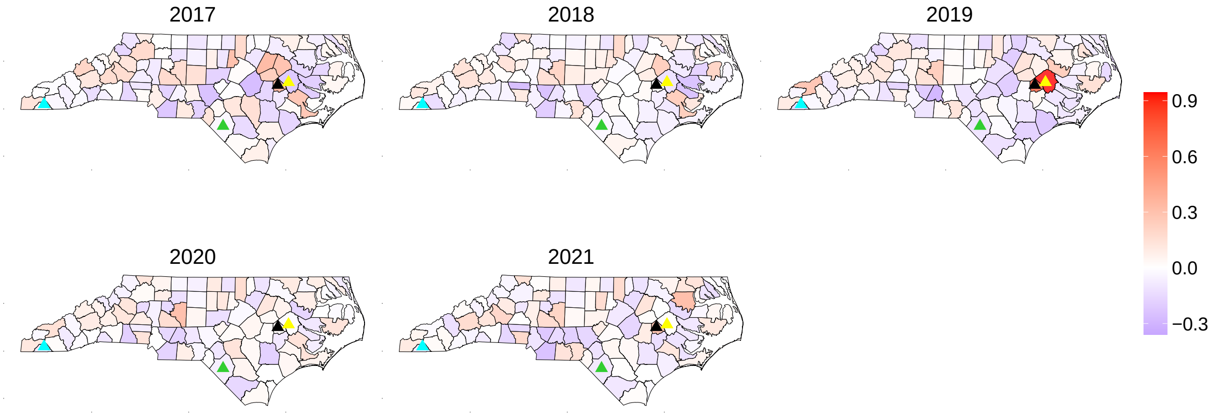

Fig. SM27. Posterior mean estimated uncorrelated heterogeneity for total HCV infections from 2017 to 2021.

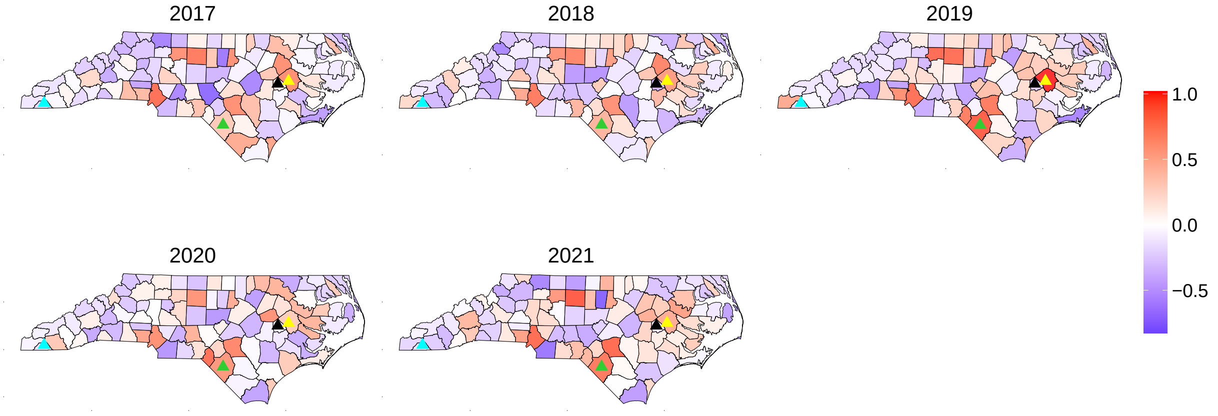

Fig. SM28. Posterior mean estimated uncorrelated heterogeneity for newly diagnosed HIV infections from 2017 to 2021.

SM 6. Factor 1 residual

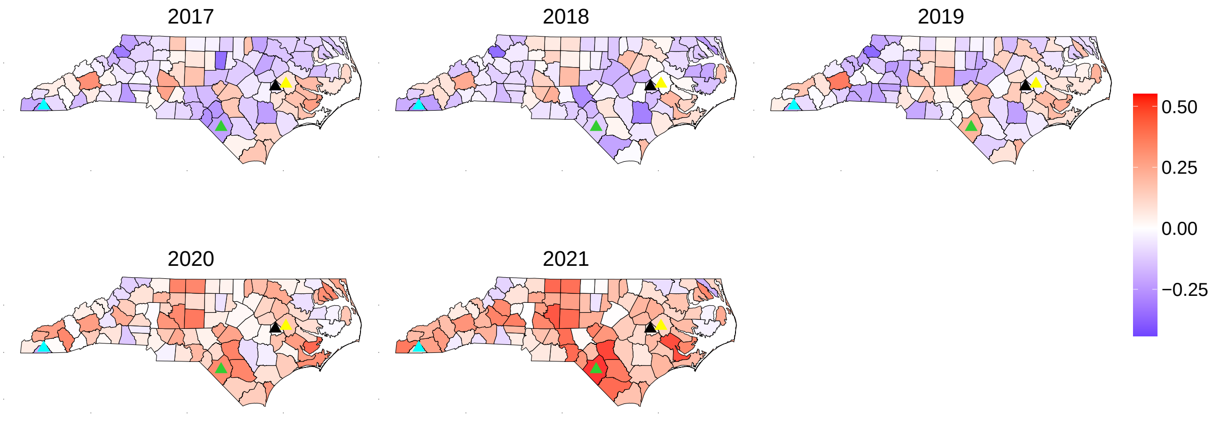

Fig. SM29. Death counts residuals after accounting for Factor 1.

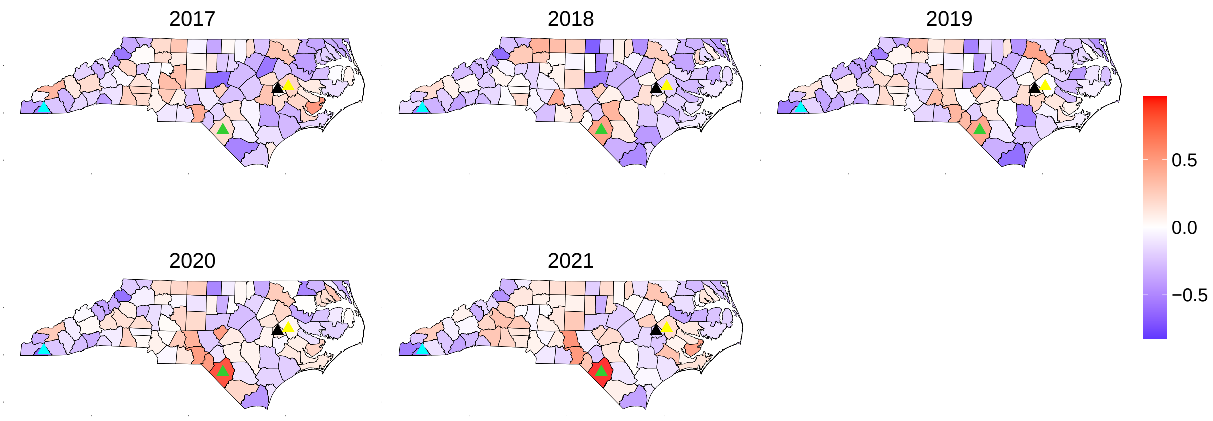

Fig. SM30. ED visits residuals after accounting for Factor 1.

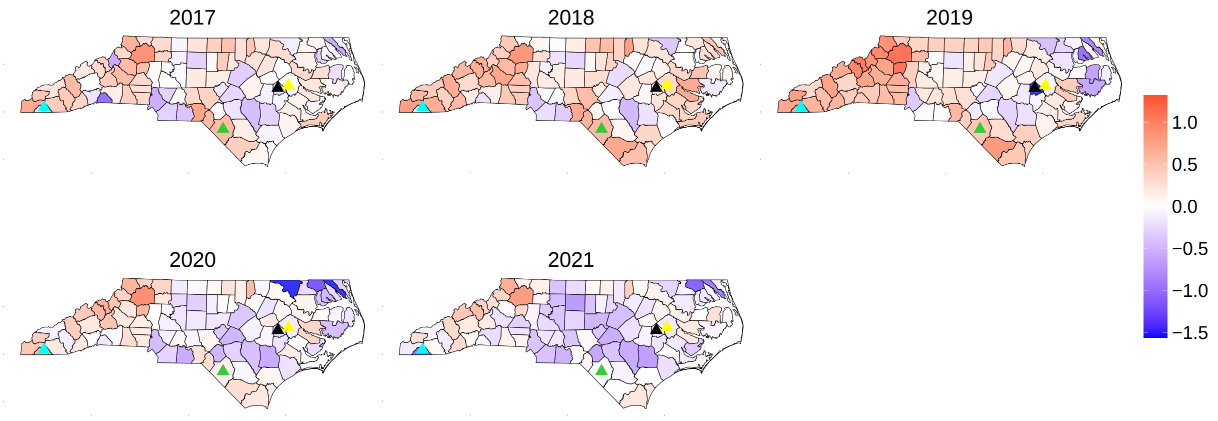

Fig. SM31. Treatment counts residuals after accounting for Factor 1.

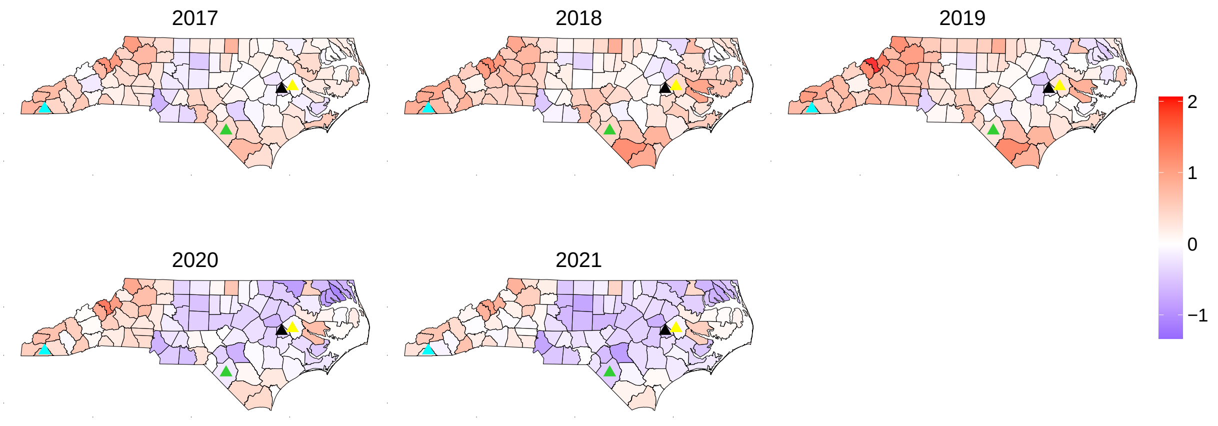

Fig. SM32. Buprenorphine prescriptions residuals after accounting for Factor 1.

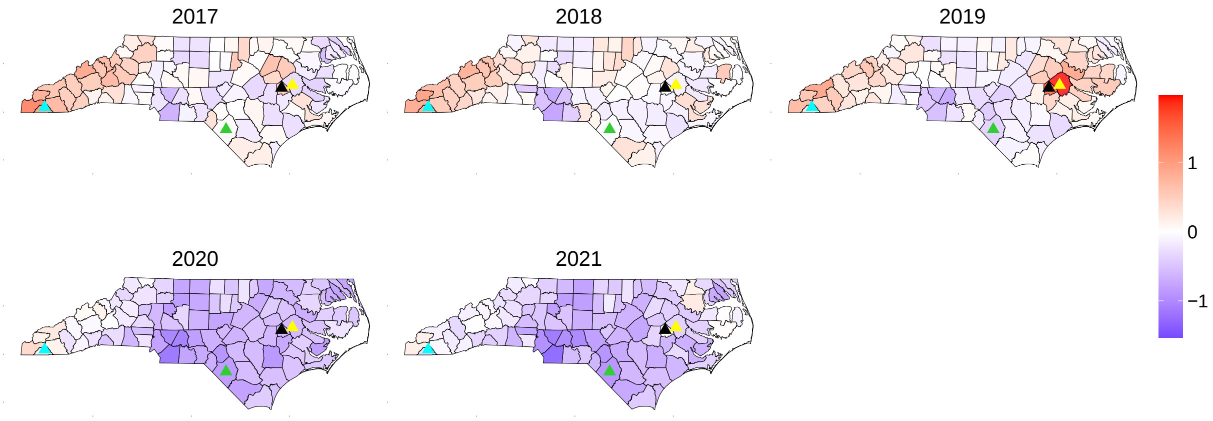

Fig. SM33. HCV infections residuals after accounting for Factor 1.

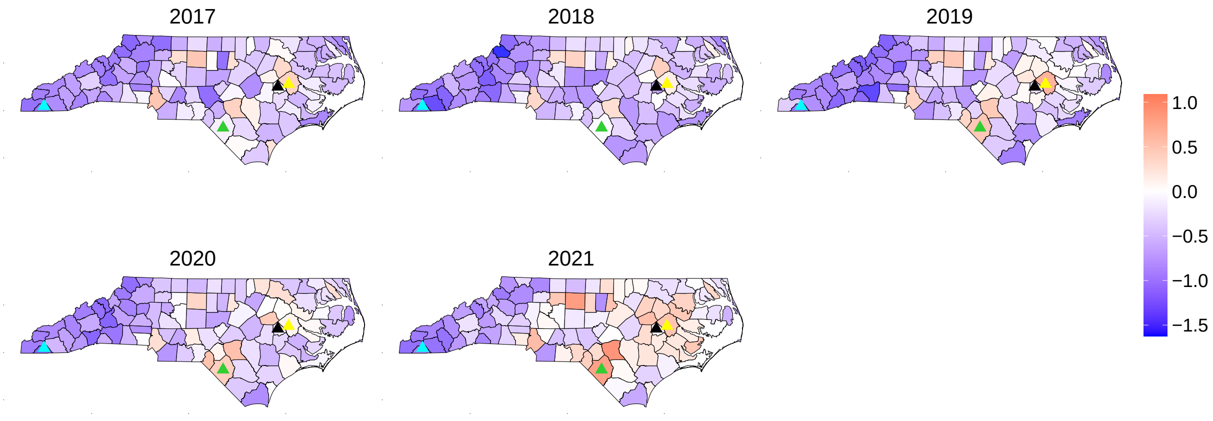

Fig. SM34. HIV infections residuals after accounting for Factor 1.

**Outcomes**	**Prop.var F1**	**Prop.var F1&F2**	**Prop.var F1&F3**	**Prop.var F1&F4**
Death	0.44	0.92	0	0
ED visits	0.24	0.68	0	0
Treatment	0.65	0	0.90	0
Buprenorphine	0.72	0	0.88	0
HCV	0.56	0	0	0.85
HIV	0.33	0	0	0.37

Table SM1. Proportion of the variability in each of the six outcomes explained by Factor 1 (**second column**), Factors 1 and 2 (**third column**), Factors 1 and 3 (**fourth column**), Factors 1 and 4 (**fifth column**).
